# Genomic regions associated with muscularity in beef cattle differ in five contrasting cattle breeds

**DOI:** 10.1186/s12711-020-0523-1

**Published:** 2020-01-30

**Authors:** Jennifer L. Doyle, Donagh P. Berry, Roel F. Veerkamp, Tara R. Carthy, Ross D. Evans, Siobhán W. Walsh, Deirdre C. Purfield

**Affiliations:** 1 Teagasc, Animal and Grassland Research and Innovation Centre, Moorepark, Fermoy, Co. Cork Ireland; 2 Department of Science, Waterford Institute of Technology, Cork Road, Waterford, Co. Waterford Ireland; 3 Animal Breeding and Genomics Centre, Wageningen University and Research Centre, Livestock Research, Wageningen, The Netherlands; 4 Irish Cattle Breeding Federation, Bandon, Co. Cork Ireland

## Abstract

**Background:**

Linear type traits, which reflect the muscular characteristics of an animal, could provide insight into how, in some cases, morphologically very different animals can yield the same carcass weight. Such variability may contribute to differences in the overall value of the carcass since primal cuts vary greatly in price; such variability may also hinder successful genome-based association studies. Therefore, the objective of our study was to identify genomic regions that are associated with five muscularity linear type traits and to determine if these significant regions are common across five different breeds. Analyses were carried out using linear mixed models on imputed whole-genome sequence data in each of the five breeds, separately. Then, the results of the within-breed analyses were used to conduct an across-breed meta-analysis per trait.

**Results:**

We identified many quantitative trait loci (QTL) that are located across the whole genome and associated with each trait in each breed. The only commonality among the breeds and traits was a large-effect pleiotropic QTL on BTA2 that contained the *MSTN* gene, which was associated with all traits in the Charolais and Limousin breeds. Other plausible candidate genes were identified for muscularity traits including *PDE1A*, *PPP1R1C* and multiple collagen and *HOXD* genes. In addition, associated (gene ontology) GO terms and KEGG pathways tended to differ between breeds and between traits especially in the numerically smaller populations of Angus, Hereford, and Simmental breeds. Most of the SNPs that were associated with any of the traits were intergenic or intronic SNPs located within regulatory regions of the genome.

**Conclusions:**

The commonality between the Charolais and Limousin breeds indicates that the genetic architecture of the muscularity traits may be similar in these breeds due to their similar origins. Conversely, there were vast differences in the QTL associated with muscularity in Angus, Hereford, and Simmental. Knowledge of these differences in genetic architecture between breeds is useful to develop accurate genomic prediction equations that can operate effectively across breeds. Overall, the associated QTL differed according to trait, which suggests that breeding for a morphologically different (e.g. longer and wider versus shorter and smaller) more efficient animal may become possible in the future.

## Background

Linear type traits have been used extensively to characterize conformation in both dairy [1–3] and beef cattle [4, 5]. Muscularity linear type traits have previously been documented as moderate to highly heritable traits in beef cattle [5–7] and are known to be genetically associated with carcass merit [8, 9] and with both animal live weight and price [4]. Therefore, the genetic merit of a young animal for these traits may be a good representation of its merit for carcass traits. While both carcass value and conformation have been reported to be correlated with linear type traits [9], the correlation with any one type trait is not equal to 1 which implies that the same carcass value can be achieved with morphologically different animals; by extension then, this implies that, for example, an animal with a better developed loin and a shallow chest may have the same yield as an animal with a lesser developed loin and a deep chest. Such morphological differences could contribute, in turn, to differences in individual carcass retail cut weights, and thus overall carcass value.

Many previous genomic studies in cattle have focused on live weight and carcass traits as the phenotypes of interest [10–12], but only a few have been published on the underlying features that contribute to differences in linear type traits in either beef cattle [13] or dairy cattle [14]. While previous studies have attempted to compare and contrast putative mutations, genes, and associated biological pathways across multiple breeds of beef cattle for carcass traits [15], no study has attempted to do this using linear type traits. Knowledge of any kind of similarities or differences between breeds could enable the introduction of more accurate multi-breed genomic evaluations for both pure and crossbred animals. Therefore, the objective of the present study was to identify genomic regions associated with five muscularity linear type traits and to determine if these associated regions are common across multiple beef cattle breeds.

## Methods

### Phenotypic data

As part of the Irish national beef breeding program, routine scoring of linear type traits is carried out on both registered and commercial beef herds by trained classifiers who are employed by the Irish Cattle Breeding Federation [4, 16], with each classifier scoring animals from a range of different breeds. The muscularity type traits used in the present study describe the development of the hind quarter (DHQ), inner thigh (DIT), and loin (DL), and the width of the thigh (TW) and withers (WOW). Each trait was scored on a scale from 1 to 15 where 1 = low and 15 = high for DHQ, DIT and DL, and 1 = narrow and 15 = wide for TW and WOW (see Additional file 1: Table S1). Data on these five linear type traits were available for 147,704 purebred Angus (AA), Charolais (CH), Hereford (HE), Limousin (LM), or Simmental (SI) beef cattle scored between the age of 6 and 16 months from 2000 to 2016 [7].

Animals were discarded from the dataset if the sire, dam, herd, or classifier was unknown, or if the parity of the dam was not recorded. Parity of the dam was recoded as 1, 2, 3, 4, and ≥ 5. Contemporary group was defined as herd-by-scoring date generated separately per breed. Each contemporary group had to have at least five records. Following these edits, data were available on 81,200 animals: 3356 AA, 31,049 CH, 3004 HE, 35,159 LM and 8632 SI.

### Generation of adjusted phenotypes

Prior to inclusion in the analysis, all phenotypes were first adjusted within-breed in ASREML [17] using the model:

$$y = HSD + Sex + AM + DP + Animal + e,$$

where *y* is the linear type trait, *HSD* is the fixed effect of herd by scoring date (11,130 levels), *Sex* is the fixed effect of the sex of the animal (male or female), *AM* is the fixed effect of the age in months of the animal (11 classes from 6 to 16 months), *DP* is the fixed effect of the parity of the dam (1, 2, 3, 4 and ≥ 5), *Animal* is the random additive effect of the animal, and *e* is the random residual effect. The adjusted phenotype was the raw phenotype minus the fixed effect solutions of *HSD*, *Sex, AM* and *DP*.

### Genotype data

Of the 81,200 animals with linear type trait information, 19,449 animals from five beef breeds (1444 AA, 6433 CH, 1129 HE, 8745 LM, and 1698 SI) were imputed to whole-genome sequence as part of a larger dataset of 638,662 multi-breed genotyped animals. All 638,662 animals were genotyped using the Bovine Illumina SNP50 panel [n = 5808; 54,001single nucleotide polymorphisms (SNPs)], the Illumina High Density (HD) panel (HD; n = 5504; 777,972 SNPs), the Illumina 3k panel (n = 2256; 2900 SNPs), the Illumina low-density (LD) genotyping panel (n = 15,107; 6909 SNPs) or a bespoke genotype panel (IDB) developed in Ireland [18] with three versions, i.e. version 1 (n = 28,288; 17,137 SNPs), version 2 (n = 147,235; 18,004 SNPs) and version 3 (n = 434,464; 53,450 SNPs). Each animal had a call rate higher than 90% and only autosomal SNPs, SNPs with a known chromosome and position on UMD 3.1, and SNPs with a call rate higher than 90% within a panel were retained for imputation.

All genotyped animals were imputed to HD using a two-step approach in FImpute2 with pedigree information [19]; this involved imputing the 3 k, LD and IDB genotyped animals to the Bovine SNP50 density, and consequently imputing all resulting genotypes (including the Bovine SNP50 genotypes) to HD using a multi-breed reference population of 5504 influential sires genotyped on the HD panel. Imputation to whole-genome sequence (WGS) was then undertaken using a reference population of 2333 *Bos taurus* animals from multiple breeds from Run6.0 of the 1000 Bull Genomes Project [20]. All variants in the sequence reference population were called using SAMtools and genotype calls were improved using the Beagle software to provide a consensus SNP density across all animals. Details of the alignment to UMD 3.1 bovine reference genome, variant calling and quality controls completed within the multi-breed reference population are described in Daetwyler et al. [20]. In total, 41.39 million SNPs were identified across the genome and the average coverage was 12.85X. Imputation of the HD genotypes to WGS was completed by first phasing all 638,662 imputed HD genotypes using Eagle (version 2.3.2) [21], and subsequently imputing to WGS using minimac3 [22]. The average genotype concordance of imputation to WGS, defined as the proportion of correctly called SNPs versus all SNPs using a validation set of 175 Irish animals, was estimated to be 0.98 [23].

Quality control edits were imposed on the imputed sequence genotypes within each breed, separately. Regions of poor WGS imputation accuracy, which could be due to local mis-assemblies or mis-orientated contigs, were removed. These regions were identified using an additional dataset of 147,309 verified parent progeny relations as described by [23], which removed 687,352 SNPs from each breed. Then, all SNPs with a minor allele frequency (MAF) lower than 0.002 were removed. Following all SNP edits, 16,342,970, 17,733,147, 16,638,022, 17,803,135 and 17,762,681 autosomal SNPs remained for the analysis of the AA, CH, HE, LM, and SI populations, respectively.

### Association analyses

The association analyses were performed within each breed separately using a linear mixed model in the GCTA software [24]. Autosomal SNPs from the original HD panel (i.e., 734,159 SNPs) were used to construct the genomic relationship matrix (GRM). The model used for the within-breed analysis was the following:

$${\mathbf{y}} = \mu + {\mathbf{xb}} + {\mathbf{u}} + {\mathbf{e}},$$

where **y** is a vector of preadjusted phenotypes, *μ* is the overall mean, **x** is the vector of imputed genotypes, **b** is the vector of additive fixed effects of the candidate SNP to be tested for association, ${\mathbf{u}}\sim N\left( {{\mathbf{0}},{\mathbf{G}}\upsigma_{\text{u}}^{2} } \right)$ is the vector of additive genetic effects, where **G** is the genomic relationship matrix calculated from the HD SNP genotypes and $\upsigma_{\text{u}}^{2}$ is the additive genetic variance, and ${\mathbf{e}}\sim N\left( {{\mathbf{0}},{\mathbf{I}}\upsigma_{\text{e}}^{2} } \right)$ is the vector of random residual effects and $\upsigma_{\text{e}}^{2}$ is the residual variance. Manhattan plots were created for each trait within each breed separately by using the QQman package [25] in R.

### QTL detection, gene annotation and variance explained

A genome-wide SNP significance threshold of p ≤ 1 × 10^−8^ and a suggestive threshold of p ≤ 1 × 10^−5^ were applied to each trait. SNPs in close proximity to each other (< 500 kb) were classified as being located within the same QTL. Genes within 500 kb of the most significant SNP in a peak above the genome-wide threshold were identified using Ensembl 94 [26] on the UMD 3.1 bovine genome assembly. Moreover, the functional consequence of all significantly associated SNPs was predicted using the Variant Effect Predictor tool [27] from Ensembl. The Cattle QTLdb (https://www.animalgenome.org/cgi-bin/QTLdb/BT/index) was used to identify QTL that were known to be associated with other traits in cattle. To identify QTL regions that were suggestive in more than one breed, each chromosome was split into 1-kb genomic windows, and windows containing suggestive SNPs (p ≤ 1 × 10^−5^) were compared across the breeds.

The proportion of genetic variance of a trait explained by a SNP was calculated as:

$$\frac{{2p\left( {1 - p} \right)a{}^{2}}}{{\upsigma_{\text{g}}^{2} }},$$

where *p* is the frequency of the minor allele, *a* is the allele substitution effect and $\upsigma_{\text{g}}^{2}$ is the genetic variance of the trait in question.

### Meta-analysis

Following the within-breed association analyses, meta-analyses were conducted for all traits across all five beef breeds using the weighted *Z*-score method in METAL [28]; only SNPs that were included in the analyses of all of the individual breeds were considered here. METAL combines the p-values and the direction of SNP effects from individual analyses, and weights the individual studies based on the sample size to compute an overall *Z*-score:

$$Z = \frac{{\varSigma_{i} z_{i} w_{i} }}{{\sqrt {\varSigma_{i} w_{i}^{2} } }},$$

where *w*_i_ is the square root of the sample size of breed *i*, and *z*_i_ is the *Z*-score for breed *i* calculated as $z_{i} = \phi^{ - 1} \left( {1 - \frac{{p_{i} }}{2}} \right)\Delta_{i}$, where *ϕ* is the cumulative distribution function, and *P*_i_ and Δ_i_ are the p-value and direction of effect for breed *i*, respectively.

### Conditional analyses

The summary statistics from the individual analyses for the CH population were further used to conduct conditional analyses on BTA2 based on the Q204X mutation, which was previously reported to be associated with muscularity traits in cattle [29]. These analyses were undertaken for each trait in the CH population using the conditional and joint association analysis (COJO) method in GCTA [30]. The Q204X mutation was included as a fixed effect in the association analysis model and the allele substitution effect of all remaining SNPs were re-estimated.

### Pathway and enrichment analyses

Pathway analysis was conducted on all plausible candidate genes within a 500-kb region up- and downstream of SNPs that were discovered to be suggestively or significantly associated with each trait in each breed. For each gene list, DAVID 6.8 [31] was used to identify gene ontology (GO) terms and KEGG pathways which were significantly overrepresented (p < 0.05) by the set of genes. Enrichment analyses among the suggestive and significant SNPs were performed to estimate if the number of SNPs in each annotation class was greater than that expected by chance for each trait per breed [32]; this was done separately per trait and per breed and was calculated as:

$${\text{Enrichment}} = \frac{{\text{a}}}{{\text{b}}}\left[ {\frac{{\text{c}}}{{\text{d}}}} \right]^{{ - 1}},$$

where ${\text{a}}$ is the number of suggestive and/or significant SNPs in the annotation class of interest, ${\text{b}}$ is the total number of suggestive and/or significant SNPs that were associated with the trait of interest, ${\text{c}}$ is the total number of SNPs in the annotation class in the association analysis, and ${\text{d}}$ is the overall number of SNPs included in the association analysis.

## Results

Summary statistics of the five linear type traits for each breed are in Additional file 1: Table S1. Significant (p ≤ 1 × 10^−8^) and/or suggestive (p ≤ 1 × 10^−5^) SNPs were detected in all traits for the five breeds but the exact locations of these SNPs and the direction of the effects of these SNPs differed by breed. Manhattan plots for all the analyses are available in Additional file 2: Figures S1–S5.

### Within-breed analyses

#### Angus

Whereas no significant SNPs were detected for any of the muscularity linear type traits in the AA population, suggestive SNPs (p ≤ 1 × 10^−5^) were identified for all five traits. No genomic region was common to all five type traits (see Additional file 3: Figure S6). However, there was some overlap in suggestive 1-kb windows between the traits DIT and TW; 11 windows contained SNPs of suggestive significance and the gene *EMILIN22* on BTA24 was identified within those windows for both traits. Nine genomic windows were associated with both the DL and WOW traits, i.e. on BTA6 (n = 2), BTA15 (n = 6), and BTA22 (n = 1). The windows on BTA15 contained suggestive SNPs that were located within the *UCP3* and *CHRDL2* genes.

Eighty-four SNPs within nine QTL were suggestively associated with the DHQ trait. Among these, the most strongly associated (p = 3.34 × 10^−7^) SNP was rs433492843 on BTA23 located in an intron of the *PTCHD4* gene (Table 1); it accounted for 0.002% of the genetic variance in this trait. A QTL on BTA1 was also strongly associated with DL with the most strongly associated SNP being rs465472414 (p = 1.06 × 10^−6^), which accounted for 0.08% of the genetic variance in this trait (Table 2). Other SNPs suggestively associated with DL were also identified within the *TMEM178A* gene on BTA11 and within the *UCP3* and *CHRDL2* genes on BTA15.

**Table 1:** Location of the most significant QTL, limited to the top five per breed, which were associated with development of hind quarter and the genes located within these QTL within each breed

Breed	Chr	Start	End	Number of suggestive and significant SNPs	Most significant SNP	p-value	Allele frequency of + allele					Candidate genes within this QTL
							AA	CH	HE	LM	SI	
AA	1	72069526	130071811	8	120584401^a^	3.69 × 10^−6^	0.940	0.940	0.061	0.949	0.951	*DLG1*, *FAM43A*, *APOD*, *OPA1*, *OSTN*, *GHSR*
	8	72017409	73103211	16	72569526^b^	3.25 × 10^−6^	0.276	0.565	0.264	0.332	0.687	*ADAM28*
	10	5155837	6179062	11	5655837^d^	7.60 × 10^−6^	0.032	0.133	0.000	0.073	0.036	*SFXN1*, *DRD1*^e^
	23	20541063	21541072	2	21041063^b^	3.34 × 10^−7^	0.995	0.977	0.000	0.000	0.005	*PTCHD4*^e^
	24	35913368	37107434	16	36567715^a^	8.07 × 10^−7^	0.918	0.050	0.958	0.941	0.903	*ENOSF1*, *ADCYAP1*
CH	2	35194	10711228	5128	6808074^a^	9.07 × 10^−49^	0.000	0.079	0.000	0.028	0.004	*WDR75*^f^, *ASNSD1*^f^, *ARHGEF4*^f^, *MYO7B*^f^, *IWS1*^e^, *NAB1*^f^, *MFSD6*^f^, *MSTN*^f^, *PMS1*^f^, *ORMDL1*^e^, *COL3A1*^f^, *COL5A2*^f^, *ANKAR*^f^, *SLC40A1*^f^
	4	89299122	90487119	12	89799122^a^	4.67 × 10^−6^	0.000	0.007	0.002	0.000	0.992	*GPR37*^e^, *POT1*
	14	33353270	34360874	4	33855595^a^	2.20 × 10^−7^	0.013	0.026	0.054	0.054	0.982	*PREX2*
	21	34538609	35572213	47	35062974^b^	9.70 × 10^−7^	0.617	0.521	0.594	0.000	0.451	*UBL7*, *SEMA7A*, *PML*
	28	15669176	18516719	77	16273851^a^	2.95 × 10^−8^	0.574	0.827	0.580	0.416	0.520	*ANK3*, *CDK1*, *RHOBTB1*
HE	1	68813144	73614763	228	69348458^b^	5.23 × 10^−7^	0.764	0.531	0.237	0.317	0.480	*KALRN*^e^, *ITGB5*^e^
	7	83591608	84673367	26	84122153^a^	3.16 × 10^−7^	0.177	0.851	0.913	0.000	0.910	*ACOT12*, *ATG10*
	12	48965278	50129513	4	49465278^a^	1.84 × 10^−6^	0.020	0.995	0.996	0.983	0.000	*KLF12*
	13	2761800	3761897	2	3261897^a^	7.44 × 10^−7^	0.681	0.761	0.795	0.269	0.695	*MRPL33*
	23	12056019	13111217	5	12571991^b^	1.09 × 10^−6^	0.220	0.000	0.094	0.885	0.100	*GLO1*, *GLP1R*
LM	2	4293223	12640428	2610	6622189^a^	3.22 × 10^−30^	0.491	0.447	0.250	0.043	0.799	*WDR75*, *ASNSD1*^f^, *MYO7B*, *IWS1*, *NAB1*^f^, *MFSD6*^e^, *MSTN*^f^, *PMS1*^f^, *ORMDL1*^e^, *COL3A1*^f^, *COL5A2*^f^, *ANKAR*^f^, *SLC40A1*^f^, *ZNF804A*
	2	13916060	14987913	113	14446207^bc^	4.83 × 10^−8^	0.567	0.562	0.374	0.394	0.523	*PDE1A*^e^, *PPP1R1C*
	5	59612855	60696179	7	60112855^a^	1.58 × 10^−7^	0.000	0.000	0.000	0.997	0.996	*AMDHD1*
	11	12163298	13181229	7	12676690^a^	1.56 × 10^−6^	0.486	0.283	0.000	0.231	0.163	*CYP26B1*, *DYSF*, *ZNF638*
SI	6	76112104	77238886	16	76612104^a^	1.28 × 10^−6^	0.997	0.993	0.000	0.000	0.993	*ENSBTAG00000043492*
	7	103195804	104247192	3	103695804^b^	1.78 × 10^−6^	0.000	0.000	0.000	0.005	0.005	*SLCO4C1*^e^, *SLC06A1*
	9	65068999	66303927	4	65712927^a^	4.56 × 10^−7^	0.000	0.000	0.000	0.003	0.996	*TBX18*^e^, *MRAP2*
	23	41204249	42287354	9	41747427^a^	1.48 × 10^−7^	0.009	0.008	0.994	0.962	0.990	*JARID2*
	25	22649471	23937019	6	23400365^a^	1.00 × 10^−7^	0.985	0.993	0.000	0.993	0.998	*AQP8*, *ZKSCAN2*

SNP classification: ^a^intergenic, ^b^intron, ^c^upstream gene variant ^d^downstream gene variant

Significance of SNPs within genes: ^e^gene contained at least one suggestive SNP, ^f^gene contained at least one significant SNP

**Table 2:** Location of the most significant QTL, limited to the top 5 per breed which were associated with development of loin, and the genes located within these QTL within each breed

Breed	Chr	Start	End	Number of suggestive and significant SNPs	Most significant SNP	P-value	Allele frequency of + allele					Candidate genes within this QTL
							AA	CH	HE	LM	SI	
AA	1	39155170	40155196	3	39655188^a^	1.06 × 10^−6^	0.003	0.015	0.000	0.967	0.976	
	10	5741208	6752759	54	6241208^a^	3.45 × 10^−6^	0.181	0.105	0.090	0.773	0.872	*GCNT4*, *HMGCR*
	11	21539414	22560915	4	22049725^c^	6.26 × 10^−6^	0.032	0.928	0.904	0.000	0.068	*CDKL8*, *MAP4K3*
	15	53716499	55635294	27	55096343^a^	1.43 × 10^−6^	0.993	0.000	0.000	0.000	0.996	*RAB6A*, *MRPL48*, *UCP2*, *UCP3*^d^, *PPME1*, *NEU3*
	19	12894306	13934964	9	13430257^a^	2.23 × 10^−6^	0.045	0.088	0.969	0.102	0.044	*USP32*, *MYO19*, *ACACA*
CH	1	142705947	143712126	78	143205947^c^	3.50 × 10^−6^	0.000	0.010	0.005	0.972	0.993	*BACE2*, *RIPK4*, *PRDM15*, *C2CD2*
	2	37387	8714844	1728	6808074^a^	1.19 × 10^−26^	0.000	0.079	0.000	0.028	0.004	*WDR75*^e^, *ASNSD1*^e^, *MFSD6*^e^, *MSTN*^e^, *PMS1*^e^, *ORMDL1*, *COL3A1*^e^, *COL5A2*^e^, *ANKAR*^e^, *SLC40A1*^e^
	10	84923776	85960329	2	85423776^a^	4.45 × 10^−7^	0.000	0.007	0.000	0.000	0.024	*PSEN1*, *ACOT2*, *ACOT4*, *DNAL1*, *ZNF410*, *FAM161B*, *COQ6*
	16	32025893	33120555	127	32558878^a^	4.12 × 10^−7^	0.975	0.884	0.135	0.898	0.093	*SMYD3*, *KIF26B*, *EFCAB2*
	24	45933369	46937392	5	46437392^b^	1.20 × 10^−6^	0.000	0.006	0.012	0.000	0.019	*PSTPIP2*, *ST8SIA5*
HE	2	79648265	81037622	15	80168803^b^	1.42 × 10^−6^	0.000	0.994	0.996	0.987	0.997	*STAT1*, *MYO1B*^d^, *NABP1*
	4	3339555	4351559	4	3851559^a^	1.16 × 10^−7^	0.014	0.034	0.025	0.884	0.952	*ENSBTAG00000044810*
	11	17934758	18942324	3	18442324^a^	1.11 × 10^−6^	0.876	0.011	0.011	0.964	0.988	*CRIM1*
	16	75812761	76823259	3	76312761^a^	1.94 × 10^−6^	0.911	0.000	0.030	0.000	0.937	*ENSBTAG00000044497*
	29	9233633	10275049	26	9733633^a^	1.80 × 10^−6^	0.650	0.417	0.304	0.378	0.801	*EED*, *SYTL2*, *CREBZF*, *TMEM126A*, *TMEM126B*
LM	2	5545383	8287013	748	6747317^a^	6.69 × 10^−10^	0.198	0.409	0.617	0.073	0.511	WDR75^d^, ASNSD1^d^, MFSD6, MSTN^d^, PMS1^d^, ORMDL1, *COL3A1*, *COL5A2*^d^, *ANKAR*^e^, *SLC40A1*^d^
	3	99009887	100073842	5	99509887^b^	5.78 × 10^−7^	0.041	0.000	0.047	0.934	0.883	*CYP4X1*, *CYP4A22*
	5	71738658	72751064	6	72238658^b^	2.70 × 10^−6^	0.000	0.819	0.163	0.251	0.830	*SYN3*, *TIMP3*, *MGC137211*, *MGC137014*, *LARGE1*^d^
	6	110629080	112106706	11	111176155^a^	3.30 × 10^−7^	0.126	0.868	0.898	0.830	0.211	*HS3ST1*
	12	32803688	33961156	7	33461156^b^	5.12 × 10^−7^	0.000	0.000	0.000	0.997	0.975	*USP12*, *SHISA2*
SI	7	58665425	60243933	10	59590500^a^	3.37 × 10^−7^	0.002	0.016	0.046	0.000	0.990	*SH3RF2*^d^
	8	90813081	91857894	14	91313081^a^	5.24 × 10^−7^	0.957	0.627	0.064	0.346	0.291	*SPIN1*, *FBXW12*
	14	79527472	81070931	18	80091780^b^	3.45 × 10^−7^	0.908	0.052	0.981	0.114	0.070	*E2F5*
	17	69082585	70268366	4	69646862^b^	1.04 × 10^−7^	0.975	0.010	0.987	0.992	0.003	*PITPNB*^d^
	22	33231032	34644069	14	34044822^b^	9.77 × 10^−9^	0.000	0.960	0.946	0.000	0.004	*FAM19A1*, *SUCLG2*^e^, *KBTBD8*

SNP classification: ^a^intergenic, ^b^intron, ^c^downstream gene variant

Significance of SNPs within genes: ^d^gene contains at least one suggestive SNP, ^e^gene contains at least one significant SNP

An intergenic SNP located on BTA29, rs109229230, was the most strongly associated (p = 1.82 × 10^−7^) with DIT (Table 3). Ninety-eight SNPs were suggestively associated with TW. The strongest QTL association with TW was on BTA13, on which 10 SNPs of suggestive significance were identified in a 1-Mb region (Table 4); rs137458299 displayed the strongest association (p = 2.99 × 10^−7^) and explained 0.9% of the genetic variation in TW. One hundred and seventy-three SNPs were associated with WOW in the AA population; among these 29.4% were located on BTA14 (Table 5) and the most strongly associated SNP, rs468048676, (p = 2.34 × 0^−9^), was an intergenic variant on BTA6.

**Table 3:** Location of the most significant QTL, limited to the top 5 per breed, which were associated with development of inner thigh, and the genes located within these QTL within each breed

Breed	Chr	Start	End	Number of suggestive and significant SNPs	Most significant SNP	P-value	Allele frequency of + allele					Candidate genes within this QTL
							AA	CH	HE	LM	SI	
AA	1	146687440	147685998	7	147187440^b^	9.55 × 10^−7^	0.988	0.712	0.073	0.179	0.631	*TRAPPC10*, *COL18A1*, *SLC19A1*, *PCBP3*^e^, *COL6A1*, *COL6A2*
	4	69229353	70999401	38	70373241^a^	4.19 × 10^−7^	0.991	0.015	0.990	0.014	0.900	*HOXA1*, *HOXA2*, *HOXA3*, *HOXA4*, *HOXA5*, *HOXA6*, *HOXA7*, *HOXA9*, *HOXA10*, *HOXA11*, *HOXA13*
	24	36998437	38030390	4	37530390^bd^	2.24 × 10^−7^	0.977	0.000	0.014	0.036	0.983	*NDC80*, EMILIN2^e^, *MYOM1*, *MYL12A*, *MYL12B*
	25	35042698	36122096	6	35542698^a^	3.50 × 10^−7^	0.998	0.000	0.000	0.000	0.002	*POLR2J*, *MYL10*, *ALKBH4*, *COL26A1*
	29	23787949	24826548	7	24290699^a^	1.82 × 10^−7^	0.048	0.043	0.937	0.939	0.919	*SLC6A5*, *PRMT3*
CH	2	7850	10711228	5075	6808074^a^	9.07 × 10^−49^	0.000	0.079	0.000	0.028	0.005	WDR75^f^, *ASNSD*1^f^, *NAB1*^f^, *MFSD6*^f^, *MSTN*^f^, *PMS1*^f^, *ORMDL*^e^ *COL3A1*, *COL5A2*^f^, *ANKAR*^f^, *SLC40A1*^f^
	14	33353270	34360874	4	33855595^a^	2.20 × 10^−7^	0.013	0.026	0.054	0.054	0.018	*ARFGEF1*, *CPA6*, *PREX2*
	14	67849241	68850085	4	6834924^a^	2.69 × 10^−6^	0.000	0.062	0.000	0.000	0.000	*STK3*, *KCNS2*, *POP1*, *RPL30*, *MATN2*
	16	60443313	62320499	4	60943313^a^	6.72 × 10^−6^	0.904	0.230	0.063	0.853	0.821	*RASAL2*, *ANGPTL1*, *TOR3A*, *ABL2*, *SOAT1*
	29	21313583	22460213	38	21917306^a^	3.58 × 10^−6^	0.000	0.989	0.000	0.978	0.000	*GAS2*, *FANCF*
HE	4	3924012	5000928	3	4424012^a^	4.05 × 10^−7^	0.755	0.032	0.082	0.000	0.939	*ENSBTAG00000023806*
	7	72100887	73679002	67	72608186^b^	9.09 × 10^−7^	0.000	0.998	0.046	0.000	0.995	*EBF1*^e^, *ADRA1B*
	13	6038290	8341939	9	7003978^a^	1.84 × 10^−7^	0.000	0.000	0.002	0.004	0.000	*ESF1*, *NDUFAF5*, *FLRT3*
	14	57257740	58269158	3	57757740^a^	7.98 × 10^−7^	0.262	0.122	0.758	0.104	0.000	*TRHR*
	25	37995664	39005834	3	38505834^c^	2.68 × 10^−7^	0.160	0.928	0.097	0.056	0.071	*LMTK2*, *PMS2*, *EIF2AK1*, *USP42*
LM	2	313343	3758925	102	3226165^a^	5.94 × 10^−10^	0.907	0.032	0.997	0.066	0.057	*NIPA1*, *NIPA2*^e^, *ARHGEF4*
	2	4973733	11101064	2441	6747317^a^	2.20 × 10^−28^	0.802	0.409	0.617	0.074	0.490	*WDR75*, *ASNSD1*^f^, *NAB1*^f^, *MFSD6*^e^, *MSTN*^f^, *PMS1*^f^, *ORMDL1*^e^, *COL3A1*^e^, *COL5A2*^f^, *ANKAR*^f^, *SLC40A1*^f^
	2	11556240	12618550	36	12116324^a^	1.36 × 10^−8^	0.308	0.402	0.231	0.195	0.556	*ZNF804A*
	2	13935604	14957932	55	14447892^bc^	1.67 × 10^−6^	0.433	0.562	0.374	0.394	0.524	*PDE1A*^e^, *PPP1R1C*, *NEUROD1*
	4	23124509	24137261	59	23630609^b^	2.52 × 10^−7^	0.096	0.027	0.996	0.994	0.010	*AGMO*^e^, *MEOX2*
SI	1	22558133	23622641	49	23117106^a^	5.21 × 10^−6^	0.949	0.043	0.866	0.060	0.870	*ENSBTAG00000046369*
	14	79492849	80610485	17	80090294^b^	2.33 × 10^−7^	0.104	0.052	0.980	0.886	0.072	*E2F5*
	17	21615210	22651417	8	22115210^a^	1.47 × 10^−6^	0.474	0.619	0.639	0.359	0.297	*ENSBTAG00000044703*
	21	17057917	18336523	18	17836523^a^	5.30 × 10^−8^	0.003	0.010	0.000	0.000	0.997	*ENSBTAG00000045960*
	22	33706576	34737653	10	34236342^b^	2.08 × 10^−6^	0.000	0.995	0.000	0.987	0.002	*SUCLG2*^e^, *KBTBD8*

SNP classification: ^a^intergenic, ^b^intron, ^c^upstream gene variant, ^d^downstream gene variant

Significance of SNPs within genes: ^e^gene contains at least one suggestive SNP, ^f^gene contains at least one significant SNP

**Table 4:** Location of the most significant QTL, limited to the top 5 per breed, which were associated with thigh width, and the genes located within these QTL within each breed

Breed	Chr	Start	End	Number of suggestive and significant SNPs	Most significant SNP	P-value	Allele frequency of + allele					Candidate genes within this QTL
							AA	CH	HE	LM	SI	
AA	11	53741952	54849531	2	54349531^a^	2.65 × 10^−6^	0.017	0.047	0.000	0.997	0.994	*CTNNA2*
	13	78082912	79223584	10	78722523^b^	2.99 × 10^−7^	0.191	0.232	0.866	0.157	0.108	*ZNFX1*, *B4GALT5*, *SLC9A8*^f^, *UBE2V1*
	14	22612620	23619374	3	23116129^a^	1.99 × 10^−6^	0.026	0.994	0.074	0.962	0.971	*OPRK1*, *ATP6V1H*, *RGS20*
	16	1654734	2669694	4	2169248^a^	5.14 × 10^−6^	0.986	0.980	0.985	0.056	0.979	*ETNK2*, *GOLT1A*, *PPP1RI5B*, *PIK3C2B*, *NFASC*
	16	63066185	64073838	2	63066185^d^	4.07 × 10^−6^	0.027	0.948	0.991	0.072	0.000	*ACBD6*, *STX6*, *MR1*
CH	2	7850	10186234	1860	6808074^a^	4.09 × 10^−25^	0.000	0.079	0.000	0.028	0.004	*WDR75*^g^, *ASNSD1*^g^, *NAB1*^g^, *MFSD6*^g^, *MSTN*^g^, *PMS1*^g^, *ORMDL1*, *COL3A1*^g^, *COL5A2*^g^, *ANKAR*^g^, *SLC40A1*^g^
	9	12470065	13731582	11	12970065^e^	8.65 × 10^−9^	0.000	0.013	0.000	0.003	0.000	*KHDC3L*^g^, *EEF1A1*
	20	61727533	63246384	5	62296495^b^	7.78 × 10^−9^	0.997	0.010	0.000	0.000	0.000	*CTNND2*^f^, *DAP*, *FAM173B*
	28	9234253	24087892	1025	16275379^a^	4.17 × 10^−9^	0.581	0.829	0.476	0.430	0.523	*ANK3*^f^, *CDK1*, *RHOBTB1*, *EGR2*
	28	24331178	40249741	1322	26134688^a^	3.87 × 10^−9^	0.388	0.810	0.424	0.591	0.486	*SIRT1*, *MYPN*^f^, *DNA2*^f^, *SLC25A16*, *SRGN*^f^, *COL13A1*^f^, *AIFM2*, *ADAMTS14*^f^, *SGLP1*, *PCBD1*, *SPOCK2*^f^, *ANAPC16*^f^, *DDIT4*, *MYOZ1*
HE	5	17326996	18595093	3	18095093^a^	1.75 × 10^−6^	0.000	0.996	0.003	0.985	0.000	*C5H12orf50*, *C5H12orf29*, *CEP290*
	7	75039465	75039465	2	75539465^d^	4.24 × 10^−6^	0.990	0.000	0.997	0.000	0.000	*GABRA6*^f^
	10	87480123	88487031	3	87980123^b^	1.72 × 10^−6^	0.959	0.034	0.020	0.981	0.033	*TTLL5*^f^,* TGFB3*
	20	35840468	37412173	5	36340468^a^	1.30 × 10^−6^	0.342	0.589	0.421	0.230	0.328	*LIFR*, *EGFLAM*, *GDNF*
	20	39512041	41162914	10	40272197^b^	1.01 × 10^−6^	0.015	0.985	0.985	0.994	0.003	C1QTNF3, ADAMTS12^f^
LM	2	4973607	10670961	1526	7772897^a^	2.88 × 10^−15^	0.617	0.571	0.000	0.116	0.642	*WDR75*^f^, *ASNSD1*^g^, *NAB1*^f^, *MFSD6*^f^, *MSTN*^f^, *PMS1*^f^, *ORMDL1*, *COL3A1*, *COL5A2*, *ANKAR*^g^, *SLC40A1*^g^
	2	11570479	12640428	43	12083258^a^	1.01 × 10^−7^	0.561	0.205	0.057	0.097	0.809	*ZNF804A*
	2	13916060	14952210	55	14450953^b^	1.92 × 10^−6^	0.434	0.565	0.374	0.394	0.477	*PDE1A*^f^, *PPP1R1C*
	9	36716988	37719425	4	37216988^a^	6.31 × 10^−7^	0.245	0.649	0.547	0.247	0.286	*HS3ST5*
	27	35767035	36785436	7	36267035^c^	8.03 × 10^−6^	0.057	0.000	0.998	0.997	0.000	*SFRP1*, *GOLGA7*, *GPAT4*, *ANK1*
SI	9	32996000	34105290	5	33604527^b^	4.82 × 10^−7^	0.969	0.980	0.981	0.985	0.019	*NEPN*, *GOPC*
	14	76746937	78193017	13	77271966^a^	1.65 × 10^−7^	0.712	0.107	0.052	0.916	0.085	*MMP16*^f^, *SLC2A5*
	24	17511696	18515510	3	18015356^a^	8.99 × 10^−7^	0.461	0.449	0.299	0.506	0.378	*ENSBTAG00000045320*, *ENSBTAG00000011094*
	26	35041033	36071133	3	35541033^a^	1.97 × 10^−6^	0.000	0.006	0.004	0.994	0.995	*AFAP1L2*, *ABLIM1*, *ATRNL1*
	29	47398869	48688066	30	47898869^a^	5.42 × 10^−7^	0.790	0.197	0.886	0.850	0.108	*CCND1*, *FGF19*, *FGF4*

SNP classification: ^a^intergenic, ^b^intron, ^c^upstream gene variant, ^d^downstream gene variant, ^e^synonymous gene variant

Significance of SNPs within genes: ^f^gene contains at least one suggestive SNP, ^g^gene contains at least one significant SNP

**Table 5:** Location of the most significant QTL, limited to the top 5 per breed, which were associated with width of withers, and the genes located within these QTL within each breed

Breed	Chr	Start	End	Number of suggestive and significant SNPs	Most significant SNP	P-value	Allele frequency of + allele					Candidate genes within this QTL
							AA	CH	HE	LM	SI	
AA	1	40642399	41670931	3	41142399^b^	2.49 × 10^−6^	0.997	0.998	0.009	0.991	0.000	*EPHA6*^d^
	6	90992982	92064889	10	91513217^a^	2.34 × 10^−9^	0.008	0.019	0.009	0.995	0.005	*EREG*, *AREG*, *RCHY1*
	14	9414135	10414890	9	9914135^b^	3.85 × 10^−6^	0.016	0.936	0.144	0.969	0.951	*TG*, *KCNQ3*^d^
	14	17744843	18889304	16	18244843^b^	1.59 × 10^−6^	0.957	0.007	0.977	0.000	0.977	*ANXA13*, *KLHL38*, *FBXO32*
	15	66035436	67113172	4	66535436^b^	3.42 × 10^−7^	0.004	0.996	0.930	0.003	0.992	*APIP*, *PDHX*
CH	2	218127	8714844	1227	6808074^a^	2.02 × 10^−21^	0.000	0.079	0.000	0.028	0.004	*WDR75*^e^, *ASNSD1*^d^, *NAB1*^e^, *MFSD6*^e^, *MSTN*^e^, *PMS1*^e^, *ORMDL1*, *COL3A1*^e^, *COL5A2*^e^, *ANKAR*^e^, *SLC40A1*^e^
	4	63740415	65180447	6	64680447^a^	3.32 × 10^−7^	0.000	0.003	0.000	0.000	0.991	*NT5C3A*, *KBTBD2*
	14	67870173	68870239	7	68370173^a^	2.98 × 10^−7^	0.028	0.060	0.965	0.053	0.024	*STK3*, *KCNS2*, *POP1*, *RPL30*, *MATN2*
	28	15669176	17454059	6	16943776^a^	1.52 × 10^−8^	0.640	0.868	0.525	0.690	0.729	*ANK3*, *CDK1*, *RHOBTB1*
	28	31765108	33148059	7	32634467^a^	1.61 × 10^−7^	0.367	0.889	0.664	0.511	0.468	*KCNMA1*
HE	7	63750754	64814905	15	64309256^b^	1.46 × 10^−7^	0.022	0.949	0.008	0.011	0.009	*RPS14*, *MYOZ3*, *ZNF300*, *GPX3*^d^, *ANXA6*
	11	89579990	90599173	5	90079990^a^	5.13 × 10^−6^	0.664	0.280	0.163	0.800	0.217	*RNF144A*, *RSAD2*
	18	11547513	12549350	5	12047513^a^	4.85 × 10^−6^	0.479	0.565	0.507	0.532	0.446	*GSE1*, *IRF8*, *FOXC2*
	20	25429449	27124758	9	25929649^a^	3.38 × 10^−6^	0.532	0.000	0.559	0.000	0.480	*FST*
	26	32810942	33810987	3	33310942^b^	2.78 × 10^−6^	0.000	0.000	0.998	0.000	0.000	*GPAM*, *ACSL5*
LM	2	5547713	8495179	725	6622189^a^	8.77 × 10^−12^	0.490	0.447	0.250	0.043	0.800	*WDR75*, *ASNSD1*^d^, *NAB1*, *MFSD6*, *MSTN*, *PMS1*^d^, *ORMDL1*, *COL3A1*, *COL5A2*^e^, *ANKAR*^d^, *SLC40A1*^e^
	2	9053737	10527711	26	9559686^a^	1.53 × 10^−6^	0.464	0.504	0.213	0.127	0.456	*ITGAV*, *ZC3H15*
	2	13916060	14957655	68	14450953^b^	6.10 × 10^−8^	0.433	0.564	0.626	0.394	0.479	*PDE1A*^d^, *PPP1R1C*, *NEUROD1*
	2	20539841	21539862	3	21039862^a^	6.85 × 10^−10^	0.000	0.000	0.000	0.998	0.000	*HOXD1*, *HOXD3*, *HOXD4*, *HOXD9*, *HOXD10*, *HOXD11*, *HOXD12*, *HOXD13*
	6	19014612	21121181	13	19817910^a^	8.15 × 10^−9^	0.000	0.000	0.008	0.995	0.000	*NPNT*, *GSTCD*^d^
SI	1	79028842	80104503	3	79604503^b^	3.81 × 10^−7^	0.022	0.040	0.000	0.964	0.004	*LPP*^d^
	4	57566849	58584434	5	58084434^d^	6.35 × 10^−8^	0.902	0.261	0.457	0.248	0.229	*IMMP2L*^d^, *LRRN3*
	9	32996000	34471196	16	33604527^b^	5.67 × 10^−8^	0.969	0.979	0.019	0.985	0.019	*NEPN*, *GOPC*^d^
	12	28061051	29073791	3	28561051^d^	7.49 × 10^−7^	0.008	0.020	0.084	0.016	0.998	*PDS5B*
	20	65137216	66168943	4	65668943^a^	1.76 × 10^−7^	0.000	0.009	0.000	0.977	0.007	*FASTKD3*, *ADCY2*^d^

SNP classification: ^a^intergenic, ^b^intron, ^c^downstream gene variant

Significance of SNPs within genes: ^d^gene contains at least one suggestive SNP, ^e^gene contains at least one significant SNP

#### Hereford

No significant SNPs were detected for any of the muscularity linear type traits in the HE population, although suggestive SNPs were identified for all five traits. However, no genomic window was common to all five type traits (see Additional file 3: Figure S6); six 1-kb windows i.e. on BTA5 (n = 1), BTA7 (n = 4), and BTA25 (n = 1) were shared between DHQ and DIT with three 1-kb regions on BTA20 shared between DIT and TW.

Three hundred and eleven SNPs were suggestively associated with DHQ. The strongest association with DHQ was located within a 1-Mb QTL on BTA7 where 26 SNPs of suggestive significance were identified (Table 1). The intergenic SNP, rs446625612 (p = 1.16 × 10^−7^) was the most strongly associated with DL and located within a QTL on BTA4 encompassing the *ENSBTAG00000044810* gene. Most interestingly, the strongest association within the QTL on BTA2 with DL was an intronic variant, which explained 0.7% of the genetic variance and was located within the muscle related gene *MYO1B*.

In total, 155 SNPs were suggestively or significantly associated with DIT, and 43% of these were located within a 1-Mb QTL on BTA7 (Table 3) where a number of significant SNPs were located within the *EBF1* gene. For TW, four putative candidate genes were identified (Table 4): *GABRA6* on BTA7, *TTLL5* on BTA10, and both *ADAMTS12* and *GDNF* on BTA20. The SNP, rs380761563, which displayed the strongest association with WOW, explained 1% of the genetic variance and was located in an intron of the gene *TNIP1* on BTA7 (Table 5).

#### Charolais

There were 483 1-kb suggestive genomic windows common to all five type traits in the CH population (see Additional file 3: Figure S6), among which the vast majority (n = 482) were located on BTA2 in a region encompassing the *MSTN* gene. The final region that was shared between all five traits was on BTA11. More overlaps were found for DHQ and DIT with 904 windows being common to just these two traits, 146 windows common to DHQ, DIT, and DL, 304 windows common to DHQ, DIT, DL, and TW, and 178 windows common to DHQ, DIT, and TW. The majority of all these windows were also located on BTA2.

For each of the muscularity linear traits, we identified a QTL on BTA2 in the CH population. DHQ had the largest number of associated SNPs, i.e. 3707 suggestive and 1851 significant SNPs (Table 1), all of which were located on BTA2 within a single QTL between positions 0.35 and 9.79 Mb. In total, 41 genes including *MFSD6*, *MSTN*, and *MYO7B* were located in this QTL. For DIT, a 10-Mb QTL on BTA2 was identified that contained 5075 SNPs, of which 1796 had a p-value that met the significance threshold (Table 3), whereas 178 SNPs on BTA2 in the region between 54.1 and 86.1 Mb were significantly associated with TW (Table 4). The same SNP, an intergenic variant rs799943285, showed the strongest association with all traits. The well-known Q204X mutation within the *MSTN* gene was significantly associated with DHQ, DIT and TW, and this SNP explained 4.9, 0.05, and 0.01% of the genetic variation of each trait, respectively.

In the conditional analyses within the CH population, where the Q204X mutation was included as a fixed effect in the model, the most significant SNPs from the original analyses of each trait generally decreased in significance. The most significant SNP for all traits in the original analyses was rs799943285 (p-value ranging from 9.07 × 10^−49^ for DIT and DHQ to 2.02 × 10^−21^ for WOW). In the conditional analyses, this SNP was non-significant for DL, TW, and WOW but remained suggestive for both DIT (p = 4.02 × 10^−6^) and DHQ (p = 4.62 × 10^−6^). The most significant SNP in the conditional analyses of DHQ, DL, DIT, and TW was rs41638272, which is an intergenic SNP located 10 kb from the *SLC40A1* gene; this SNP was significant in the original analyses but its significance actually increased when the Q204X mutation was included as a fixed effect. The most significant SNP in the conditional analysis of WOW was an intergenic variant, rs457456302 (p = 4.78 × 10^−10^) that was located 0.1 Mb from the *MSTN* gene.

#### Limousin

There were 164 1-kb suggestive genomic regions that were common across all muscularity traits in the LM population (see Additional file 3: Figure S6); another 232 regions were common to the three traits DHQ, DIT, and TW, while 326 were common to just DHQ and DIT. All five traits had significant QTL located on BTA2, with four genes common to all traits located within these QTL, namely *ASNSD1*, *GULP1*, *SLC40A1*, and *ANKAR*.

For DHQ, there were 2983 SNPs above the suggestive threshold and most of these (n = 2610) were located in a single QTL on BTA2. The most significant SNP, rs211140207 (p = 3.22 × 10^−30^), was located within an 8-Mb QTL on BTA2 that contains 20 genes (Table 1). The Q204X stop-gain mutation (rs110344317) located within this QTL was significantly associated with DHQ and accounted for 2.4% of the genetic variation in this trait, although the allele frequency of the favourable mutation was only 0.02% in the LM population. The well-known *MSTN* mutation in the Limousin breed, F94L (MAF = 0.3798), did not meet the suggestive threshold for association with any of the traits. Similar to DHQ, a QTL located between 4.9 and 11 Mb on BTA2 was associated with both DIT (Table 3) and TW (Table 4). In total, 2441 and 1526 SNPs were above the suggestive threshold within this QTL on BTA2, and the variant rs110344317, which was significantly associated with DHQ, was also significantly associated with both DIT and TW. For the DL trait, 748 SNPs were suggestively associated and located between 55.4 and 82.8 Mb on BTA2. The most significant SNP associated with DL (rs379791493; p = 6.69 × 10^−10^) was also the most significantly associated SNP with DIT (p = 2.20 × 10^−28^). The most significant SNP associated with WOW, rs211140207, (p = 8.77 × 10^−12^), was an intergenic SNP that accounted for 0.4% of the genetic variance in this trait and was located in a QTL (between 5.9 and 8.4 Mb) that included 724 other significantly-associated SNPs (Table 5).

Suggestive QTL were also detected on autosomes other than BTA2 for all traits in the LM population except for DIT. A small QTL on BTA11 containing seven suggestive SNPs was associated with DHQ. The SNP with the strongest association, rs43666945 (p = 1.56 × 10^−6^), was an intergenic SNP located 2.2 Mb from the *DYSF* gene. Both DHQ and DL had suggestively associated QTL on BTA5. The most strongly associated SNP for DHQ (p = 1.58 × 10^−7^) was an intergenic SNP, rs718375830, located within a QTL between positions 59.6 and 60.6 Mb, whereas the most strongly associated SNP with DL (p = 2.70 × 10^−6^) was also an intergenic SNP, rs109909829, but was located within a QTL between 71.7 and 72.8 Mb.

#### Simmental

For the SI breed, only a few suggestive 1-kb genomic regions overlapped for more than two traits. Sixteen 1-kb windows were suggestively associated with both DHQ and DL, eight of which were located on BTA6, seven on BTA22, and one on BTA18 (see Additional file 3: Figure S6). Five 1-kb windows on BTA23 and one on BTA4 were common to both DHQ and DIT, while another 15 suggestive windows were associated with DHQ and WOW, 12 of which were located on BTA22.

The intergenic SNP, rs437686690 on BTA25, was the most strongly associated (p = 1.00 × 10^−7^) with DHQ in the SI population and accounted for 0.6% of the genetic variance in DHQ (Table 1). In total, 199 SNPs were associated with DL in the SI population, among which four met the significance threshold. The most significant SNP, rs482545354 (p = 9.77 × 10^−9^), was located in an intronic region of the *SUCGL2* gene (Table 2) on BTA22. Although 194 SNPs were suggestively associated with DIT, only one, i.e., rs798946118 (p = 5.30 × 10^−8^), achieved the significance threshold which was located on BTA21 within a 1-Mb block containing 17 other suggestive SNPs (Table 3) and accounted for 0.6% of the genetic variance of DIT. The largest 1-Mb QTL associated with TW was located on BTA29 and contained 30 suggestive SNPs (Table 4). QTL putatively associated with WOW were located on BTA1, 4, 9, 12, and 20 (Table 5) where the most significant SNP, rs801295753 (p = 5.67 × 10^−8^), was an intronic SNP on BTA9 located within both the *ROS1* and *ENSBTAG000000039574* genes.

#### Meta-analyses

Within each of the five meta-analyses (see Additional file 4), a strong association peak on BTA2 around the *MSTN* gene was detected, which is consistent with the individual association results identified in the CH and LM populations. For DIT, TW, and WOW, the most significantly associated SNP was the intergenic SNP, rs799943285 (p = 5.51 × 10^−24^), which was previously identified as the most strongly associated SNP in the CH population for each of these traits. This variant, rs799943285, was also the most significantly associated with DL in the meta-analysis, whereas the most significantly associated SNP with DHQ, rs482419628 (p = 2.06 × 10^−47^), was located further downstream on BTA2 within 5 kb of the *ASNSD1* gene.

Although the QTL on BTA2 was the most strongly associated with each of the traits analysed, we also identified several other QTL associated with muscularity. In the meta-analysis of DHQ, the most strongly associated SNP on BTA11, rs43666945 (p = 1.93 × 10^−7^), was previously identified as being associated with DHQ in the LM population, but the level of significance increased in the meta-analysis and the QTL contained three times the number of suggestive SNPs compared to that found for the LM breed only. A 1-Mb QTL on BTA7 containing the *SPRY4* and *FGF1* genes was associated with both DL and WOW in the meta-analysis; the most significant SNPs in this QTL, however, differed according to trait (see Additional file 4).

#### Enrichment of SNPs

With the exception of WOW in the AA population, intergenic SNPs were the most common annotation class of SNPs that were significantly associated with all traits in all breeds. The 3′ UTR class was enriched for all traits in the CH and LM populations, whereas there were more downstream gene variants significantly associated with DHQ and DL in the AA, CH and HE populations, and with TW in the CH, HE, and SI populations than expected by chance (Table 6). The intronic class of SNPs was enriched for all five traits in HE, for four traits (DHQ, DL, TW, and DIT) in SI, three traits in both AA (DHQ, DL, and WOW) and CH (DL, TW, and WOW) and two traits in LM (DHQ and DIT).

**Table 6:** Fold enrichment/depletion of SNPs in each annotation class for each trait in each breed

	3′ UTR variant	5′ UTR variant	Coding sequence variant	Downstream gene variant	Intergenic variant	Intron variant	Missense variant	Non-coding transcript	Splice acceptor variant	Splice region variant	Stop gained	Synonymous variant	Upstream gene variant
DHQ													
AA	–	–	–	3.76	0.89	1.02	–	–	–	–	–	–	0.73
CH	3.40	0.46	–	1.08	1.09	0.84	0.62	–	–	–	7.12	0.97	0.39
HE	1.80	–	–	4.43	0.36	2.32	–	–	–	–	–	0.91	0.49
LM	1.30	0.85	–	0.50	1.02	1.09	1.15	–	–	0.67	12.89	0.47	0.37
SI	–	–	–	0.40	0.86	1.54	–	–	–	–	–	–	0.39
DL													
AA	–	–	–	1.11	1.15	0.63	–	–	–	–	–	3.98	0.64
CH	7.36	–	–	2.10	0.96	1.00	0.72	1.60	–	–	18.71	1.07	0.50
HE	–	–	–	1.21	0.77	1.64	–	–	–	–	–	3.63	0.39
LM	2.33	–	–	0.20	1.25	0.58	–	–	79.49	–	–	–	0.19
SI	–	–	–	0.94	0.93	1.13	3.90	–	–	–	–	–	1.52
DIT													
AA	–	–	–	0.14	1.03	1.12	–	–	–	–	–	–	0.66
CH	3.47	0.48	–	0.94	1.11	0.79	0.58	–	–	–	7.47	0.96	0.40
HE	–	–	–	0.40	0.48	2.43	2.49	–	–	–	–	–	0.98
LM	1.16	1.77	99.15	0.86	0.99	1.09	2.28	–	–	0.70	13.49	0.59	0.55
SI	–	–	–	0.86	1.04	1.05	–	–	–	–	–	–	0.62
TW													
AA	–	–	–	0.64	1.19	0.64	–	–	–	–	–	2.91	0.31
CH	3.07	0.54	–	1.22	0.99	1.01	0.24	1.43	–	–	8.40	1.44	0.83
HE	–	–	–	1.10	0.87	1.50	–	–	–	–	–	–	–
LM	3.17	1.45	–	0.45	1.21	0.61	0.22	–	–	–	22.05	0.32	0.28
SI	2.10	9.65	–	3.62	0.55	1.26	0.74	–	–	23.85	–	–	5.36
WOW													
AA	3.28	–	–	0.18	0.84	1.44	–	–	–	–	–	1.75	1.67
CH	6.90	–	–	1.16	1.00	1.00	–	–	–	–	27.28	1.09	0.60
HE	–	40.89	–	1.00	0.93	1.17	–	–	–	–	–	4.95	0.52
LM	2.15	2.46	–	0.58	1.12	0.84	0.37	–	–	–	–	0.32	0.41
SI	1.99	–	–	1.35	1.12	0.65	2.79	–	–	–	–	–	1.03

#### Gene ontology and KEGG pathways

Several GO terms and KEGG pathways were over-represented by the genes identified in each analysis, although this tended to differ per breed and per trait especially in the smaller AA, HE, and SI populations. In CH and LM, five GO terms were associated with each trait: skin development (GO:0043588), collagen fibril organisation (GO:0030199), extracellular matrix structural constituent (GO:0005201), cellular response to amino acid stimulus (GO:0071230), transforming growth factor beta receptor signalling pathway (GO:0007179). One KEGG pathway, i.e. protein digestion and absorption (KEGG:map04974), was also significantly associated with all traits in CH and LM. Apart from this overlap, only a limited number of terms and pathways were over-represented across breeds. The GO term mitochondrial inner membrane (GO:0005743) was significantly over-represented for the DL trait in AA and the WOW trait in HE, although none of the same genes were significantly associated with both traits. Another GO term collagen trimer (GO:0005581) was over-represented for DIT in AA and DL in LM.

## Discussion

Whereas a number of across-breed and breed-specific pleiotropic QTL have been documented for carcass traits, birth weight, weaning weight, and mature weight in beef cattle [15], as well as for dry matter intake and growth and feed efficiency [33], no study has attempted to detect across-breed or breed-specific pleiotropic QTL for muscularity linear type traits. Previous studies have been conducted on the genetic correlations between the linear type traits themselves [7] and between both meat yield and carcass cuts with the muscularity linear type traits [34]. While these genetic correlations are moderate to strong, none is equal to 1, which implies that two animals that yield a carcass of similar merit could be morphologically different. In fact, a shorter and more muscular animal or a taller and less muscular animal could have the same total carcass weight. In turn, these animals could yield very different carcass values owing to their distribution of primal cuts. For example, the loin of an animal harbours generally the most valuable cuts [35, 36]. Therefore, selection for a better-developed loin could lead to a more valuable carcass in comparison to a carcass with a lesser-developed loin if that carcass was still within the factory specification for weight and conformation. Here, we have detected several genomic regions that are strongly associated with each of the muscularity traits analysed. However, most of these regions were unique to each trait or each breed, which indicates the existence of trait-specific and breed-specific QTL for muscularity traits. Thus, it is plausible to hypothesise that through more precise (i.e., targeting individual QTL) genome-based evaluations and selection, the morphology of an animal could be targeted to increase the output of high-quality carcass cuts and consequently improve the profitability of the farm system and the value to the meat processor [36]. While a similar conclusion could be achieved through traditional breeding means, exploiting the breed- and trait-specific QTL could be more efficient.

This is the first published genome study on muscularity linear type traits in beef cattle using sequence data and is one of the few genome-based studies that compare multiple breeds of beef cattle. The number of animals used in our study is comparable to the number of animals used in a previous across-breed comparison that focused on carcass and birth traits in 10 cattle breeds [15] and was thought to be the largest genome based-study ever performed in beef cattle at that time. This previous across-breed study was undertaken on 12 traits including birth weight, calving ease, carcass weight, and mature weight across 10 breeds and the results were similar to what we observed here for the muscularity traits. Saatchi et al. [15] identified 159 unique QTL associated with 12 traits, but only four QTL had pleiotropic effects and segregated in more than one breed. Similar results were observed in an across-breed study on dry matter intake, growth and feed efficiency in four beef cattle breeds [33]. The QTL identified for these traits were also breed-specific with little overlap among the breeds. This is comparable to our findings that show that the majority of the QTL were also trait-specific and breed-specific.

In total, approximately 83% of all QTL that are suggestively or significantly associated with a trait in our study overlapped with previously reported QTL associated with other production traits in dairy or beef cattle in the Cattle QTLdb (accessed 08 January 2019). Approximately 36% of all QTL overlapped with other traits that were specifically related to muscle in beef cattle such as body weight, carcass weight and marbling score [31], calving traits [37], Warner–Bratzler shear force [38], and longissimus muscle area [39]. One QTL on BTA17 that was associated with DIT in the SI breed was previously associated with ribeye area in a composite beef cattle breed composed of 50% Red Angus, 25% Charolais, and 25% Tarentaise [40]. Our study is further validated by the presence of significantly associated QTL regions on BTA2, which harbours the *MSTN* gene, with the five muscularity traits in the CH and LM breeds, and within the meta-analysis. In a previous study on five muscularity type traits, which were combined into one singular muscular development trait in CH, a QTL on BTA2, which contained *MSTN*, was the only region significantly associated with these traits [13].

In general, the suggestive and significant QTL, and thus genes, associated with each trait and each breed were both trait-specific and breed-specific. The low commonality of QTL among the breeds may be due to different genetic architectures underlying the traits in these breeds, or to gene-by-environment or epistatic interactions [33], or to differences in the power to detect QTL due to the large differences in population sizes between the breeds. In many cases, the significant alleles were simply not segregating in all five breeds. The differences between breeds may also be due to limitations in the imputation process with the imputation accuracy being too low to determine strong associations between a SNP and a trait; consequently, the minor suggestive associations were interpreted with caution because of the possibility of poor imputation. Overall, the largest number of overlaps among significant genes were found between the CH and LM breeds for all traits, which is not surprising considering the relative similarities in the origins of these breeds [41] and of the selection pressures they have experienced [42].

### Myostatin

*MSTN* was first observed as a negative regulator of skeletal muscle mass in mice [43] and since then has been identified as responsible for muscular hypertrophy in cattle [44, 45] and is widely known as the causal variant for many muscularity and carcass traits in cattle [46, 47]. The stop-gain mutation Q204X in *MSTN* was significantly associated with the muscularity traits in both the CH and LM populations in the present study. Previously published research showed that CH and LM calves carrying one copy of this mutated allele scored better for carcass traits than non-carrier animals and that young CH bulls carrying this mutation presented a carcass with less fat and more tender meat than non-carriers [47]. In the present study, the CH and LM animals carrying one copy of the minor allele scored significantly (p < 0.01) higher for muscularity type traits. The Q204X mutation was not significant in the AA population and it was removed during the data-editing step in both HE and SI as it was non-segregating. When Q204X was included as a fixed effect in the model for the CH animals, no SNPs located within the *MSTN* gene itself remained significant. This indicates that the significant SNPs within this gene were in tight linkage disequilibrium with Q204X, which provides evidence that this mutation may be causative for the muscularity linear type traits in the CH breed. Other genes on BTA2 that were significantly associated with some or all of the traits in CH and LM were *ORMDL1*, *PMS1*, *MFSD6*, and *NAB1*, all of which are in strong linkage disequilibrium with *MSTN* in mammals [48].

### Other candidate genes

While the major peaks on BTA2 in the analyses on CH and LM, and all the meta-analyses contain *MSTN*, a known contributor to muscle development, it is also plausible that other candidate genes within the QTL on BTA2 could also contribute to muscle development. Two such genes are *COL3A1* and *COL5A2*. Intronic variants in *COL3A1* and upstream and downstream gene variants in *COL5A2* were significantly associated with DHQ in both CH and LM; however, no SNPs within coding or non-coding regions of this gene were associated with any traits in AA, HE, or SI although the SNPs were indeed segregating. Collagen is abundant in muscle and the quantity and stability of these intramuscular fibres have previously been linked to eating palatability of beef [49]. The quantity and stability of muscle collagen are known to differ by breed [50], sex [51], and age [52] of cattle. Other collagen genes, *COL6A1*, *COL6A2*, and *COL18A1*, on BTA1 were also identified as candidate genes for DIT in the AA breed. Both type VI collagen genes have previously been linked to various muscle disorders in humans since they are known to affect muscular regeneration [53]. Type XVIII collagen has previously been proposed as a useful marker for beef marbling because it is involved in fat deposition in ruminants [54].

Another QTL on BTA2 located in the region between 13.9 and 14.9 Mb and significantly associated with four of the traits (DHQ, DIT, TW, and WOW) in the LM breed contained the *PDE1A* and *PPP1R1C* genes. The most significant SNP in this region was an intronic SNP within *PDE1A*. The *PDE1A* gene is involved in a pathway related to myofibroblast formation in smooth muscle in humans [55] while previous genome-wide studies in mice have identified the *PPP1R1C* gene as a possible candidate gene for muscle mass [56]. Overall, the allele frequencies of the favorable alleles in this 1-Mb region were similar in all five breeds, which support a breed-specific association with DHQ, DIT, TW, and WOW in LM rather than an imputation error.

An additional breed-specific QTL on BTA2 that contains numerous *HOXD* genes was associated with WOW in the LM population. The *HOXD* genes are documented as having a role in limb [57] and digit [58] formation, thus they probably also play a role in skeletal muscle development. The most significantly associated SNPs with WOW in this region were only segregating in the LM breed and had a very high favorable allele frequency (0.998) in this breed. These SNPs were fixed or very close to fixation in the four other breeds.

In the meta-analyses of DHQ, associated variants in all the breeds analysed were identified, which may be beneficial for across-breed genomic prediction [59]. Although the associations detected in the meta-analysis corresponded to associations identified in the CH and LM breeds, three of these QTL on BTA5, 11, and 12 increased in significance when compared to the within-breed analysis. The QTL on BTA5 which contained the *AMDHD1* gene, was located close to a QTL previously associated with carcass composition [43], whereas the QTL on BTA11 contains *DYSF*, a gene known to be linked with muscular dystrophy in humans [60]. The QTL on BTA14 contained the *PREX2* gene which was previously linked to carcass weight in Hanwoo cattle [61].

Interestingly, in the meta-analyses of DL and WOW, a 1-Mb QTL on BTA7 containing the *SPRY4* and *FGF1* genes became suggestively associated, although it was not associated in any breed individually. The *SPRY4* gene was reported to be associated with feed intake in cattle [62], whereas *FGF1*, a member of the fibroblast growth factor family, is thought to be involved in embryonic muscle formation [63].

Similarly, in the meta-analysis of TW, a 3-Mb QTL on BTA6 containing the *NCAPG/LCORL* genes became suggestively associated, although it was not associated in any breed individually. These genes are associated with variation in body size and height in cattle [32], humans [64], and horses [65], thus they are likely plausible candidate genes associated with muscularity linear type traits describing the size of the body.

### Gene ontology and KEGG pathways

Linear type traits are complex traits that are governed by many genes each with a small effect, and hence, are likely involved in many biological systems. Several GO terms were only associated with a single trait or a single breed; hence there was limited commonality among traits or breeds suggesting the absence of a central biological process that links these traits together. Over-represented GO terms in multiple traits and breeds include those related to skin development, collagen fibril organisation, and the transforming growth factor beta receptor signalling pathway. Each of these GO terms was associated with genes located in the large QTL on BTA2 that contained *MSTN*. Excluding the major *MSTN* QTL in these breeds, which is known to have a large effect on muscularity, the various GO terms and KEGG pathways represented by the genes associated with the muscularity traits suggest that the majority of genes identified as significantly associated with a trait are not only breed-specific but also trait-specific in many cases.

### Regulatory regions involved in the development of muscle

Although millions of SNPs were tested for association with each trait, only 79 of the SNPs suggestively or significantly associated with a trait were located in the coding region of a gene; the vast majority of the SNPs associated with the muscularity traits in any of the breeds were located outside of the coding regions. This is consistent with previous genomic studies for complex quantitative traits in cattle using HD SNP data [66] or sequence data [32]. While the coverage of the HD study [66] may not have included the coding regions required to identify significant associations within these regions, our study and a previous study on cattle stature [32] used imputed sequence data, and thus, covered the entire genome.

Whereas many studies have previously acknowledged the importance of non-coding SNPs to genetic variability, little is actually known about the mechanisms by which these SNPs contribute to variation in complex traits [67, 68]. One possibility to explain the significance of these non-coding SNPs is that the non-coding regions contain gene regulatory sequences, called enhancers, that act over long distances possibly altering the expression of a gene nearby [67]. Another possibility is that the folding of DNA into the 3-dimensional nucleus may cause distant loci, such as those in non-coding and coding regions, to become spatially close together thus enabling these regulatory regions to come into contact with genes far away or even on different chromosomes [69].

Non-coding variants such as 3′ UTR, 5′ UTR and intergenic variants were enriched for most of the traits in each breed. Downstream and upstream gene variants were also enriched in some traits. In general, the SNPs located close to and within the genes identified as candidate genes were located within non-coding or regulatory regions. For example, for DHQ in the CH breed, 60 suggestively and significantly associated SNPs were located within the *MSTN* gene; 10 of these were 3′UTR variants, 31 were downstream gene variants and 19 were intronic. Whereas regulatory regions may not have an effect on the coding sequence of any gene, they are thought to be particularly important for growth and development in humans [68, 69] and cattle [32, 70]. Thus, similar to previous observations in humans and cattle, enrichment of the non-coding classes of SNPs in our study may indicate the importance of regulatory regions for cattle muscle development.

## Conclusions

Although we identified many QTL associated with muscularity in beef cattle, our results suggest that these QTL tend to be not only trait-specific but also breed-specific. Overall, the significant SNPs contained in these QTL were more likely located in regulatory regions of genes, which suggest the importance of non-coding regions that may affect gene expression for muscle development in cattle. Some shared regions associated with muscularity were found between CH and LM, with a large-effect QTL on BTA2 containing *MSTN* being associated with the five traits analysed. This overlap between these breeds was somewhat expected, because they are subjected to similar selection pressures. Apart from this single QTL, extensive differences were observed between the breeds, which may be due to the much smaller sample sizes for AA, HE, and SI compared to the CH and LM populations that result in reduced power to detect QTL or they may be due to differences in genetic architecture of these traits among the populations. In many cases, the strongly associated SNPs in one breed were not segregating in the other breeds, and thus, were missing from the analyses. Knowledge of any potential differences in genetic architecture among breeds is important to develop accurate genomic prediction equations in across-breed analyses.

## Supplementary information

**Additional file 1: Table S1.** Number of records, and mean, and standard deviation of each linear type trait within each breed.

**Additional file 2: Figure S1.** Manhattan plots for development of hind quarters in (a) Angus, (b) Charolais, (c) Hereford, (d) Limousin, (e) Simmental, and (f) meta-analysis. **Figure S2.** Manhattan plots for development of inner thigh in (a) Angus, (b) Charolais, (c) Hereford, (d) Limousin, (e) Simmental, and (f) meta-analysis. **Figure S3.** Manhattan plots for development of loin in (a) Angus, (b) Charolais, (c) Hereford, (d) Limousin, (e) Simmental, and (f) meta-analysis. **Figure S4.** Manhattan plots for thigh width in (a) Angus, (b) Charolais, (c) Hereford, (d) Limousin, (e) Simmental, and f) Meta-Analysis. Description Manhattan plots for thigh width in each of the 5 breeds and the meta- analysis. **Figure S5.** Manhattan plots for width of withers in (a) Angus, (b) Charolais, (c) Hereford, (d) Limousin, (e) Simmental, and (f) meta-analysis.

**Additional file 3: Figure S6.** Overlapping 1-kb regions that contain at least one suggestive or significant SNP for the five muscular traits in (a) Angus, (b) Charolais, (c) Hereford, (d) Limousin and (e) Simmental. Venn Diagram of overlapping 1-kb regions containing at least one suggestive or significant SNP in each of the five breeds.

**Additional file 4.** Location of the top five most significant QTL associated with each of the traits in the meta-analysis containing all five breeds.

## Data Availability

Sequence variant genotypes were provided by participation in the 1000 Bulls consortium and a subset of the sequences can be found at NCBI BioProject PRJNA238491.
