# Supplementary material for: Genomic regions associated with muscularity in beef cattle differ in five contrasting cattle breeds
Source: Genet Sel Evol. 2020 Jan 30;52:2. doi: 10.1186/s12711-020-0523-1 (PMC6993462; doi:10.1186/s12711-020-0523-1)
Supplement: Supplementary file 1 — Additional file 1: Table S1. Number of records, and mean, and standard deviation of each linear type trait within each breed. [file 12711_2020_523_MOESM1_ESM.docx]

Table S1: The number of records, the mean, and the standard deviation of each linear type trait within each breed.

|  | Scale  1-15 | Angus | | |  | Charolais | | |  | Hereford | | |  | Limousin | | |  | Simmental | | |
| --- | --- | --- | --- | --- | --- | --- | --- | --- | --- | --- | --- | --- | --- | --- | --- | --- | --- | --- | --- | --- |
| Trait |  | n | Mean | SD |  | n | Mean | SD |  | n | Mean | SD |  | n | Mean | SD |  | n | Mean | SD |
| Development of hindquarter | low - high | 1444 | 7.71 | 1.28 |  | 6433 | 9.48 | 1.38 |  | 1129 | 7.35 | 1.34 |  | 8745 | 10.02 | 1.34 |  | 1698 | 9.31 | 1.39 |
| Development of inner thigh | low - high | 1434 | 7.47 | 1.43 |  | 6253 | 9.23 | 1.54 |  | 1128 | 7.07 | 1.51 |  | 8537 | 9.70 | 1.47 |  | 1619 | 9.02 | 1.46 |
| Development of loin | low - high | 1444 | 7.96 | 1.31 |  | 6433 | 9.61 | 1.52 |  | 1129 | 7.92 | 1.26 |  | 8745 | 9.62 | 1.47 |  | 1698 | 9.29 | 1.54 |
| Width of thigh | narrow - wide | 1444 | 7.62 | 1.37 |  | 6433 | 9.31 | 1.57 |  | 1129 | 7.38 | 1.38 |  | 8745 | 9.27 | 1.62 |  | 1698 | 9.10 | 1.57 |
| Width of withers | narrow - wide | 1440 | 8.04 | 1.56 |  | 6412 | 9.54 | 1.63 |  | 1129 | 7.80 | 1.44 |  | 8710 | 9.61 | 1.54 |  | 1682 | 9.16 | 1.74 |
