# Supplementary material for: Genomic regions associated with muscularity in beef cattle differ in five contrasting cattle breeds
Source: Genet Sel Evol. 2020 Jan 30;52:2. doi: 10.1186/s12711-020-0523-1 (PMC6993462; doi:10.1186/s12711-020-0523-1)
Supplement: Supplementary file 2 — Additional file 2: Figure S1. Manhattan plots for development of hind quarters in (a) Angus, (b) Charolais, (c) Hereford, (d) Limousin, (e) Simmental, and (f) meta-analysis. Figure S2. Manhattan plots for development of inner thigh in (a) Angus, (b) Charolais, (c) Hereford, (d) Limousin, (e) Simmental, and (f) meta-analysis. Figure S3. Manhattan plots for development of loin in (a) Angus, (b) Charolais, (c) Hereford, (d) Limousin, (e) Simmental, and (f) meta-analysis. Figure S4. Manhattan plots for thigh width in (a) Angus, (b) Charolais, (c) Hereford, (d) Limousin, (e) Simmental, and f) Meta-Analysis. Description Manhattan plots for thigh width in each of the 5 breeds and the meta- analysis. Figure S5. Manhattan plots for width of withers in (a) Angus, (b) Charolais, (c) Hereford, (d) Limousin, (e) Simmental, and (f) meta-analysis. [file 12711_2020_523_MOESM2_ESM.docx]

|  | **a)** | **b)** |
| --- | --- | --- |
| 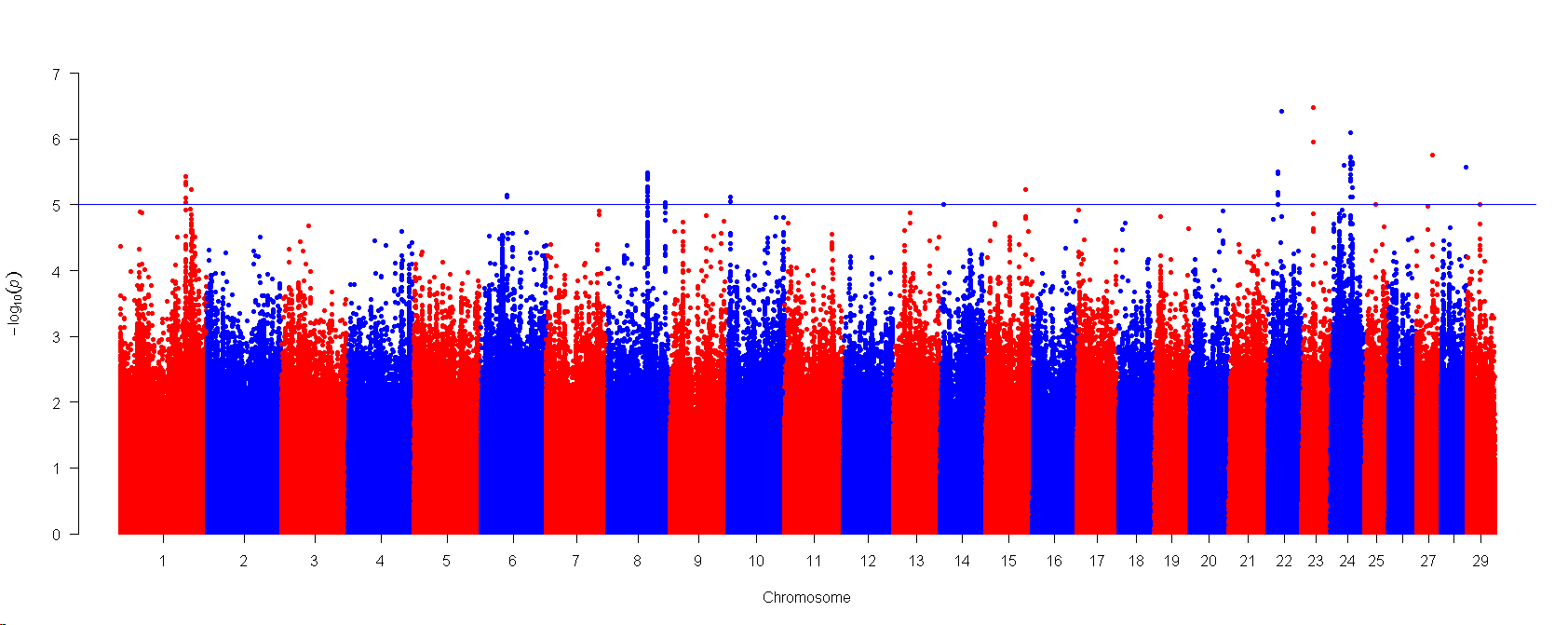 | 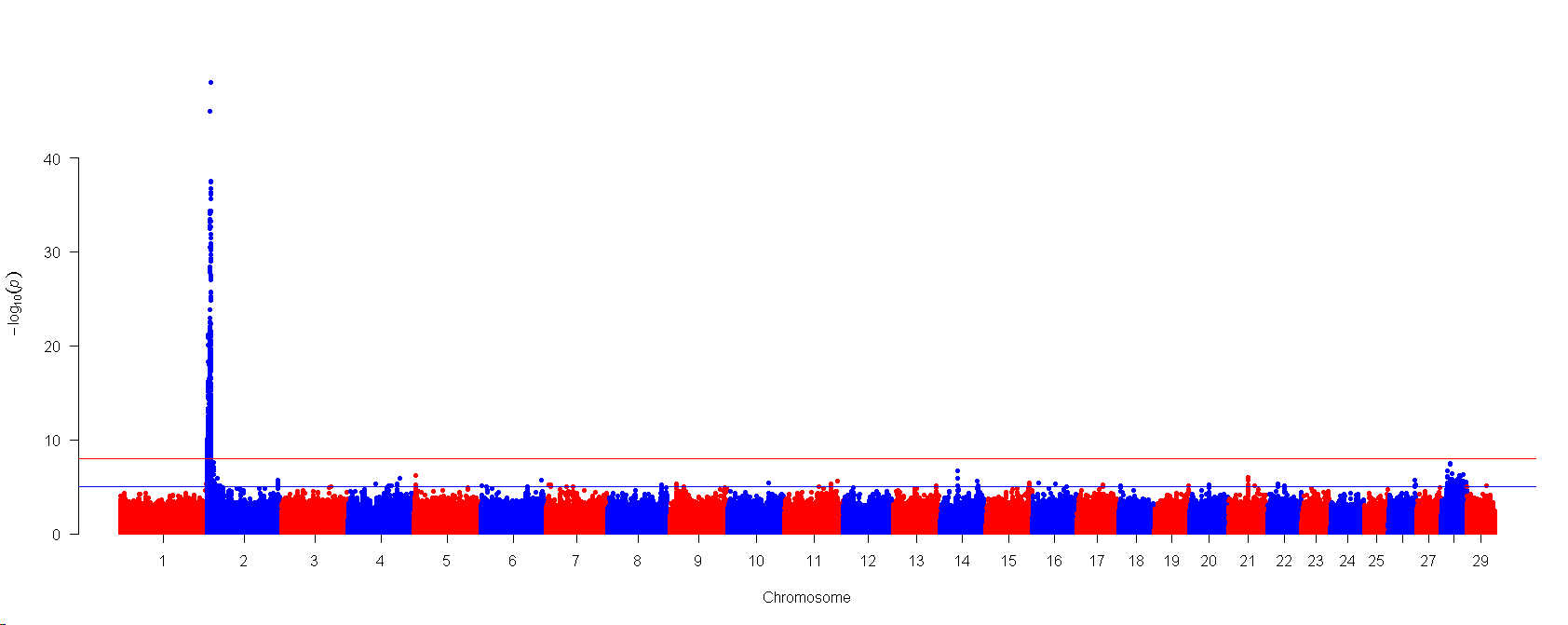 |  |
| **c)** | **d)** |  |
| 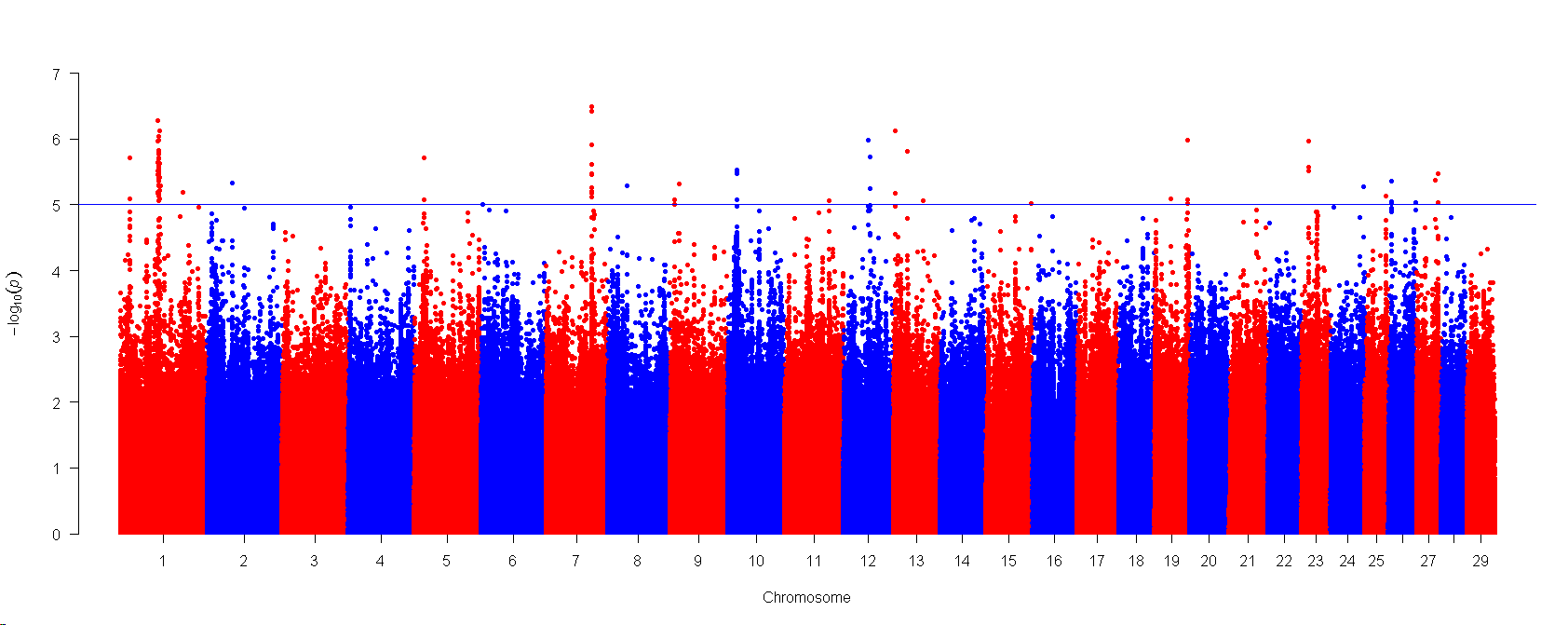 | 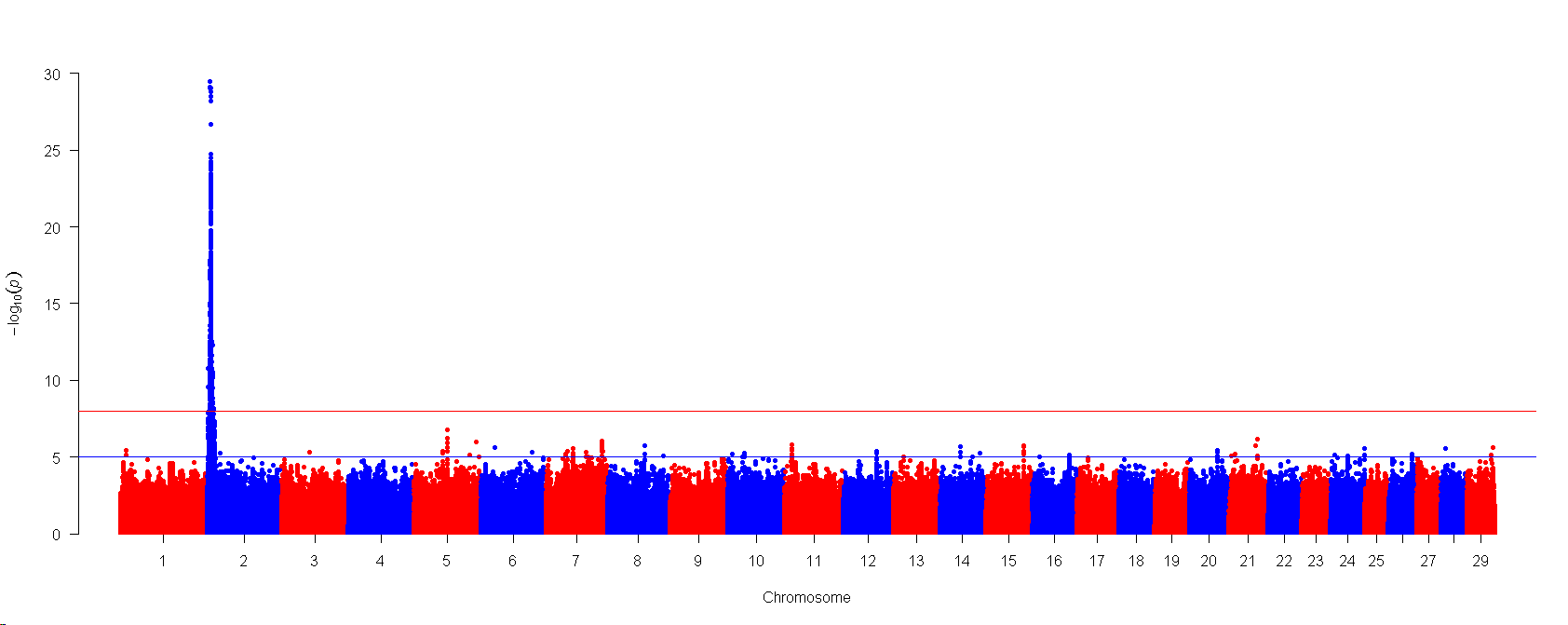 |  |
| **e)** | **f)** |  |
| 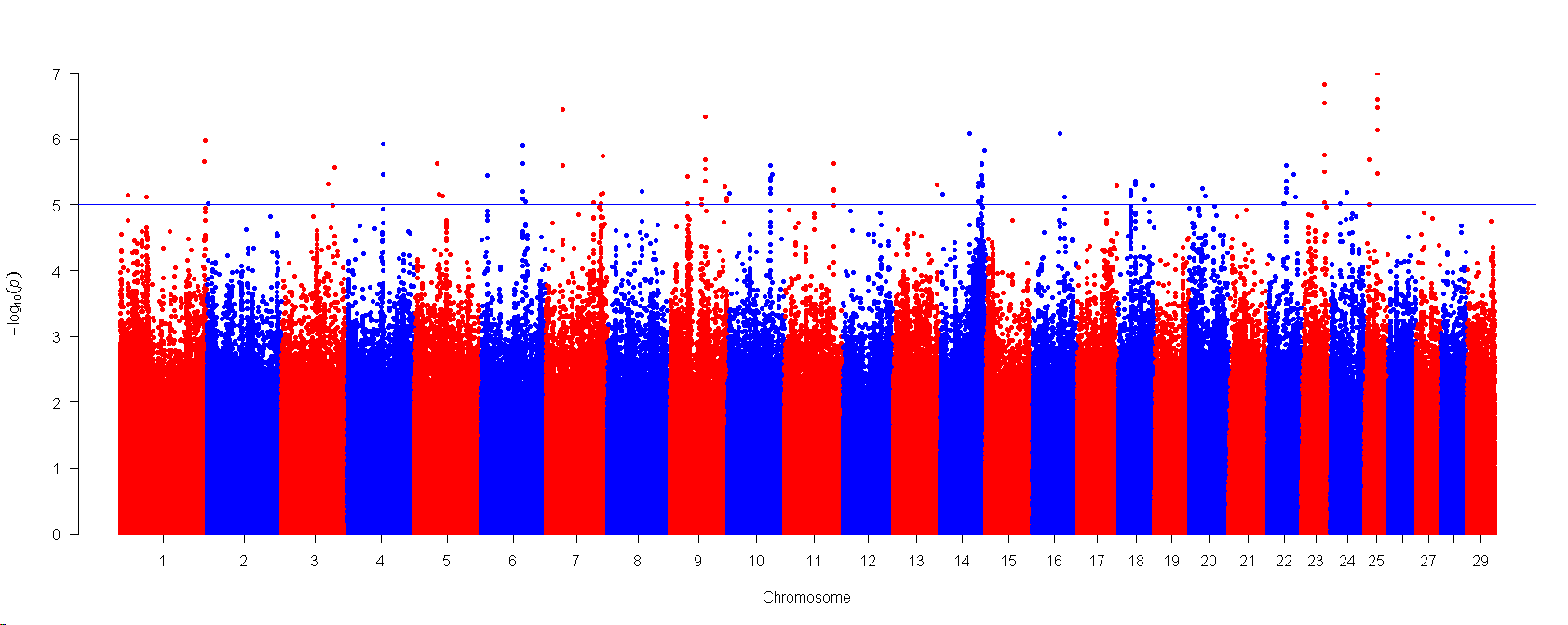 | 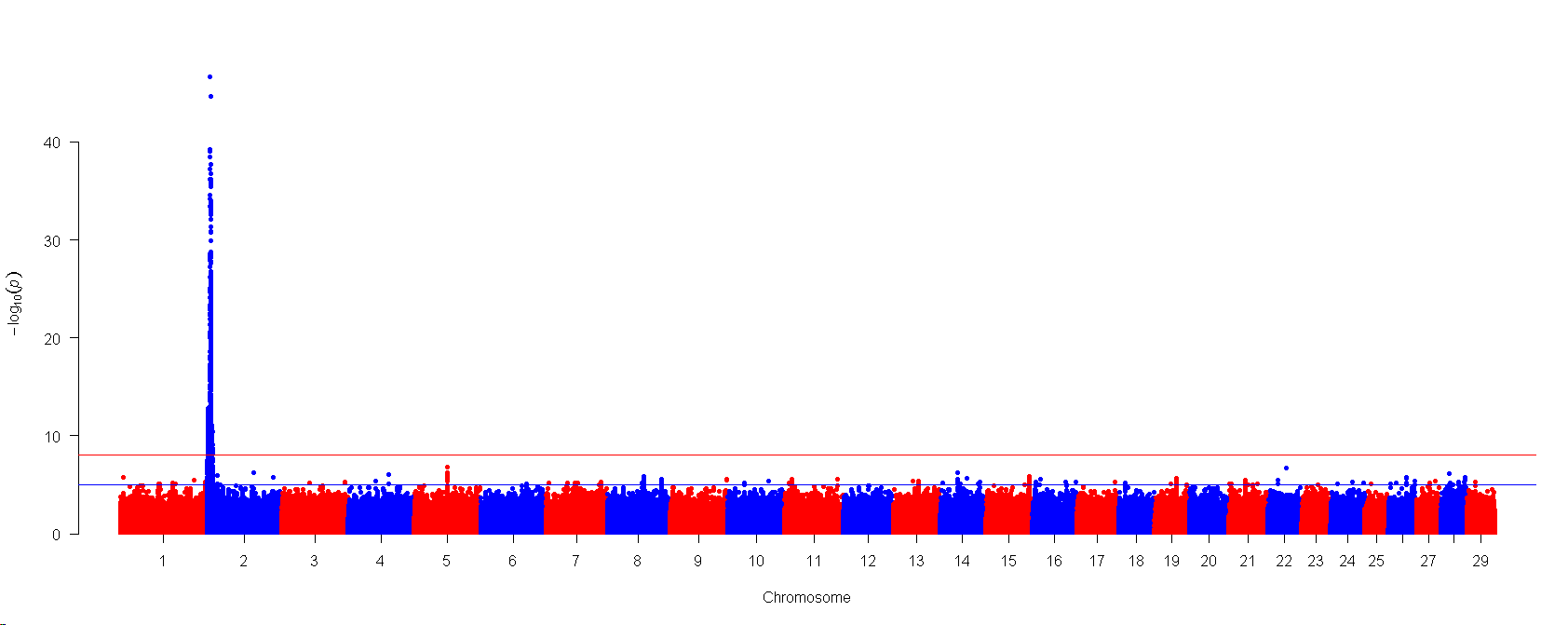 |  |

Figure S1: Manhattan plots for development of hind quarters in a) Angus, b) Charolais, c) Hereford, d) Limousin, e) Simmental, and f) Meta-Analysis.

|  | **a)** | **b)** |
| --- | --- | --- |
| 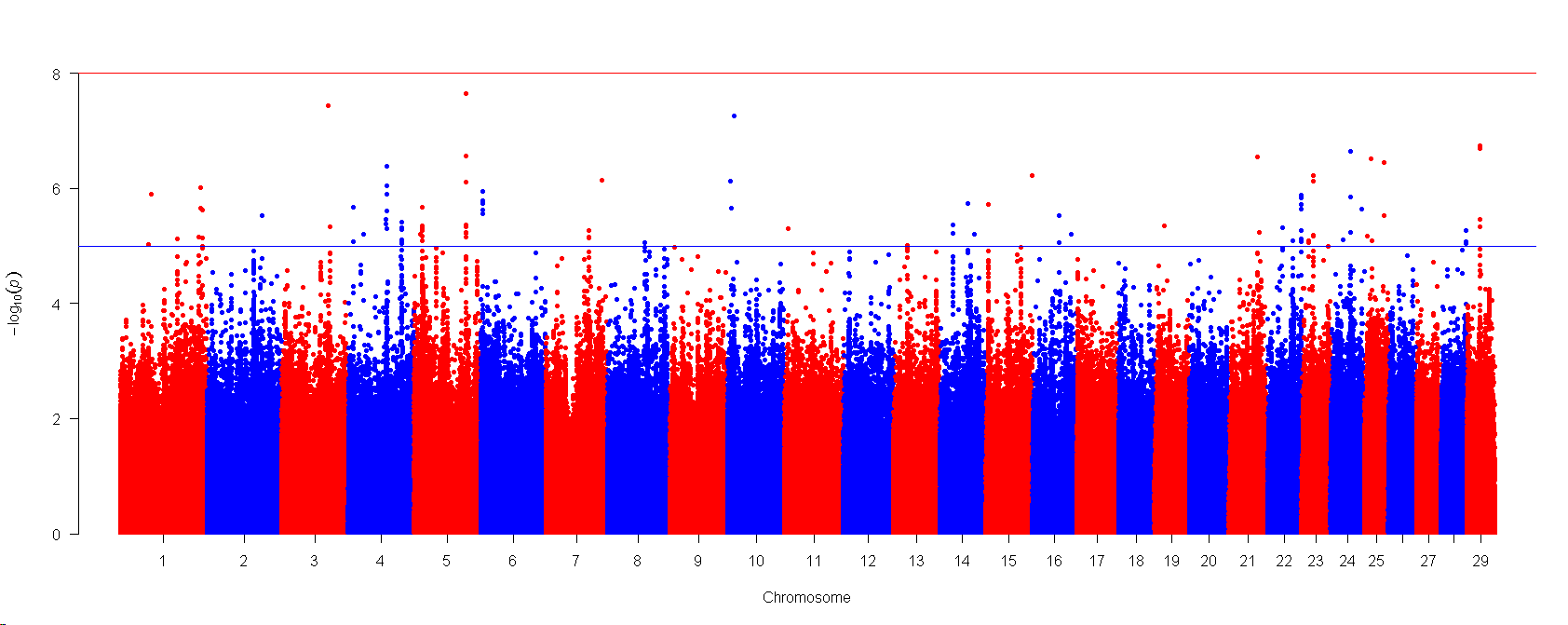 | 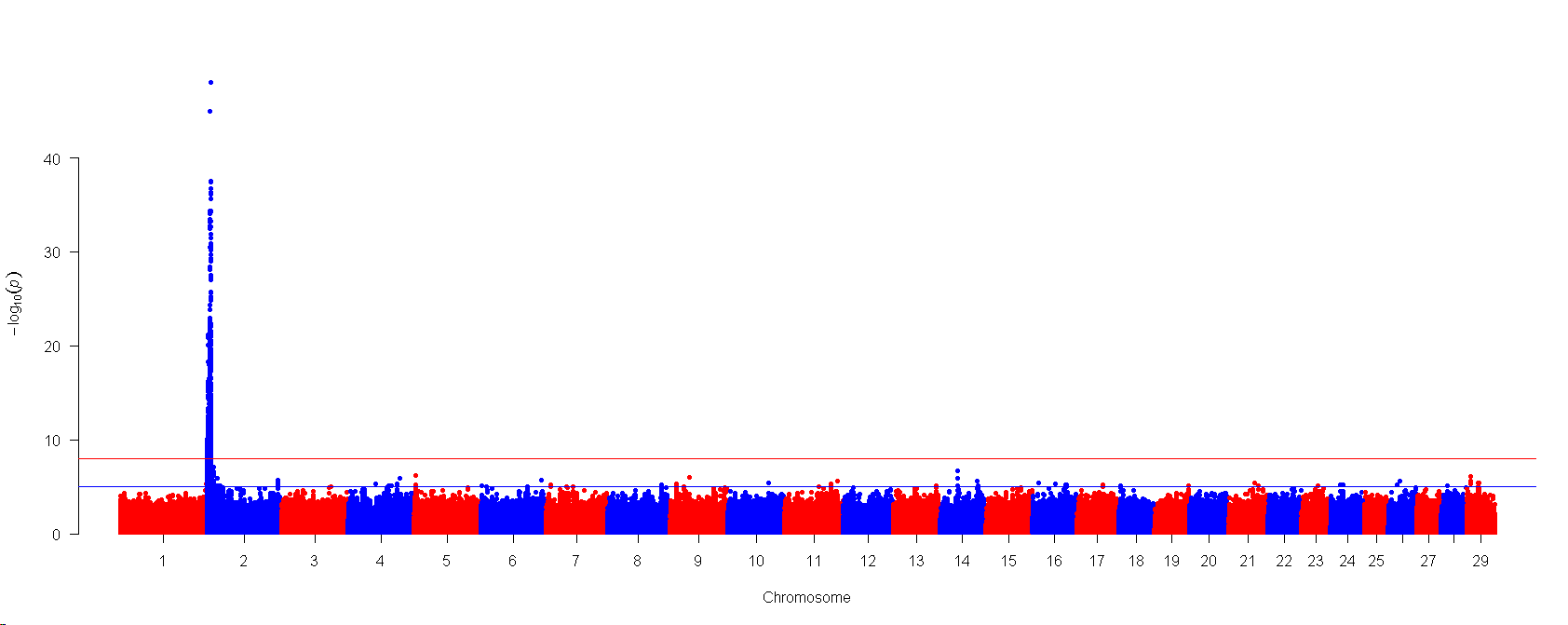 |  |
| **c)** | **d)** |  |
| 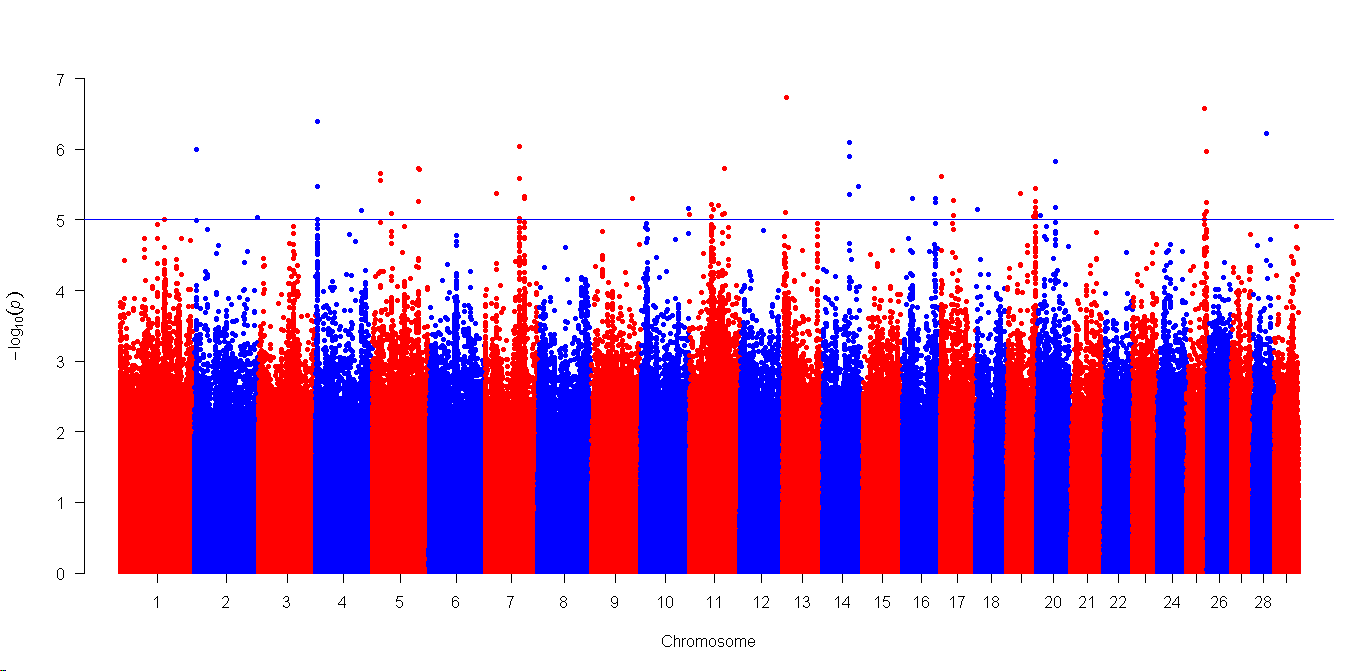 | 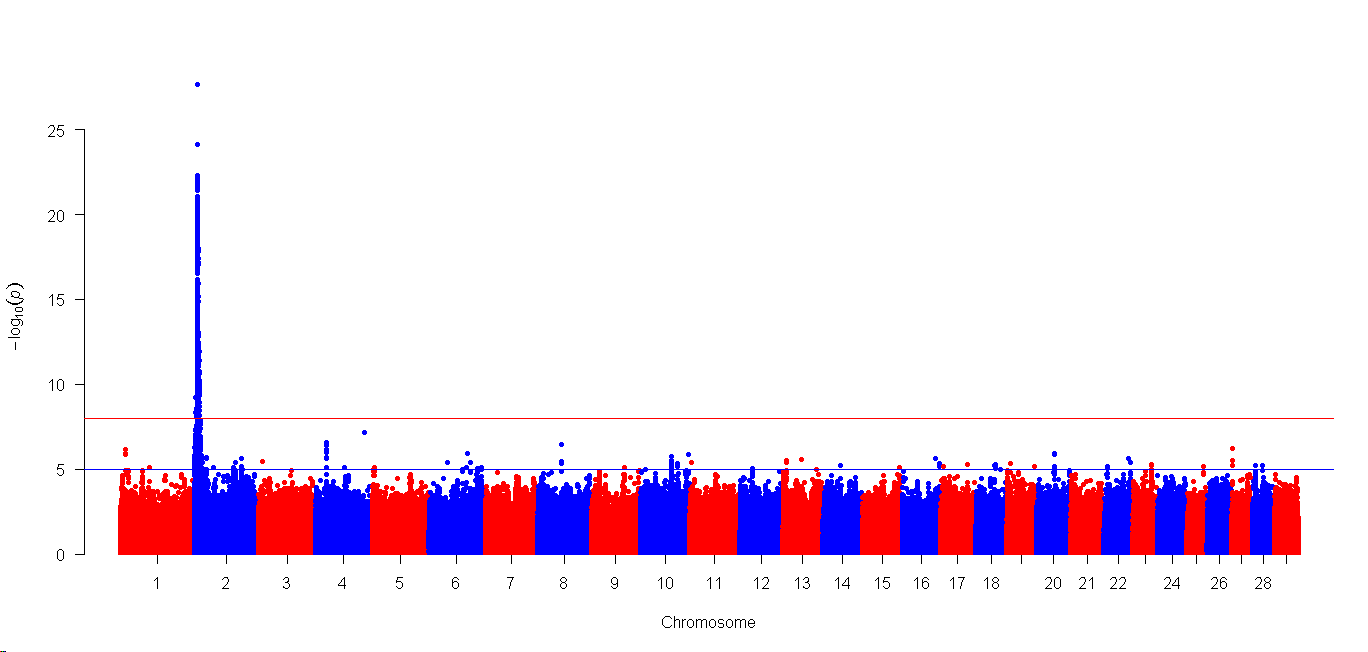 |  |
| **e)** | **f)** |  |
| 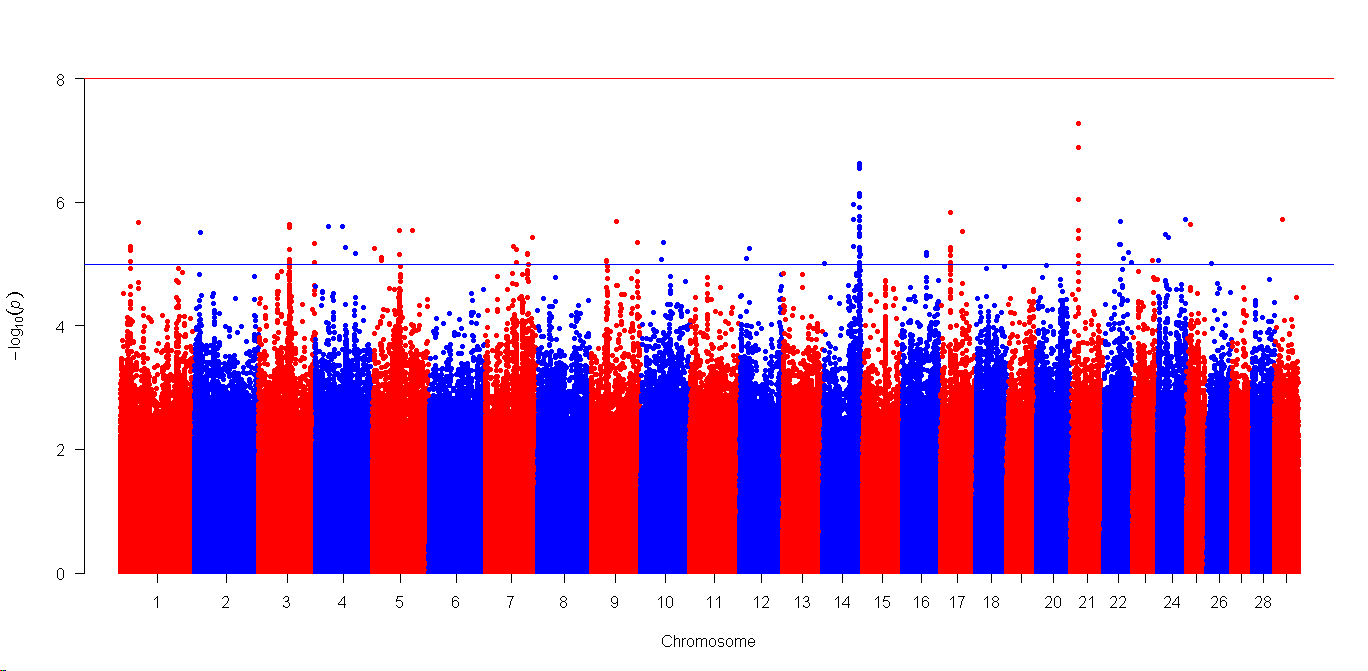 | 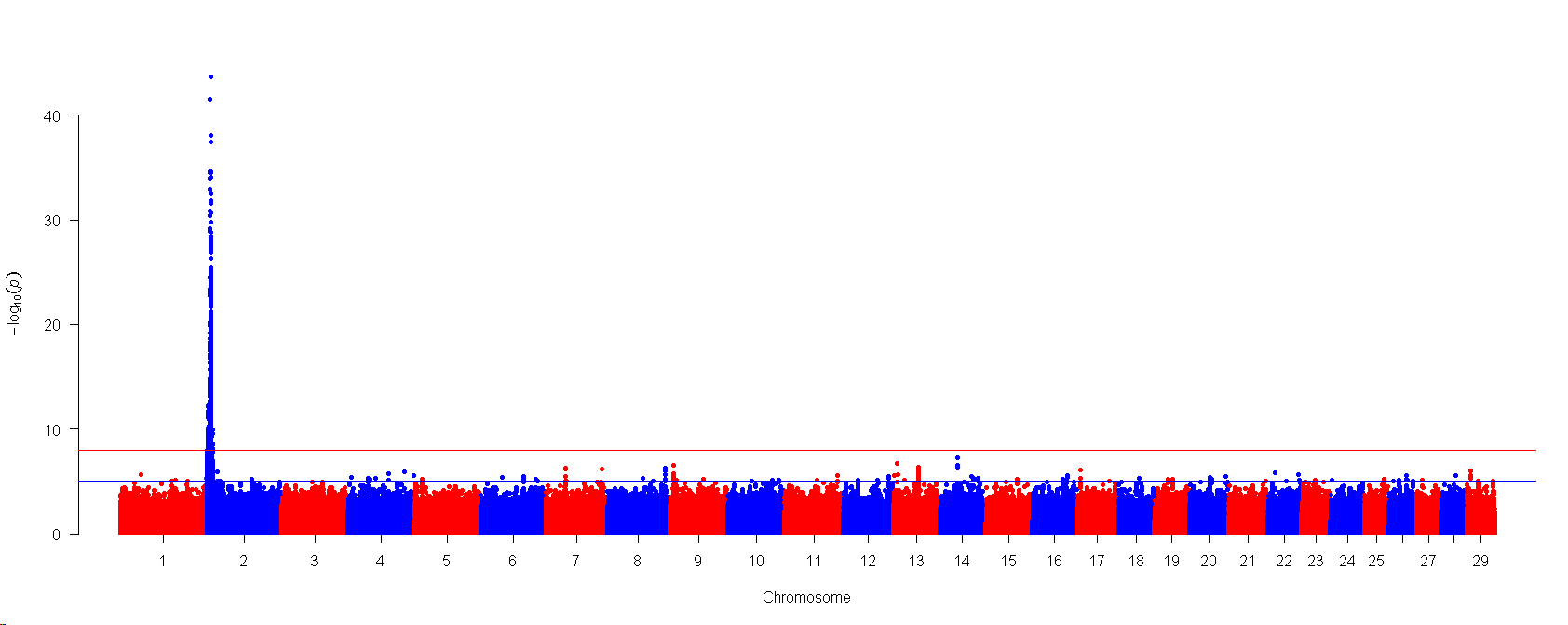 |  |

Figure S2: Manhattan plots for development of inner thigh in a) Angus, b) Charolais, c) Hereford, d) Limousin, e) Simmental, and f) Meta-Analysis.

|  | **a)** | **b)** |
| --- | --- | --- |
| 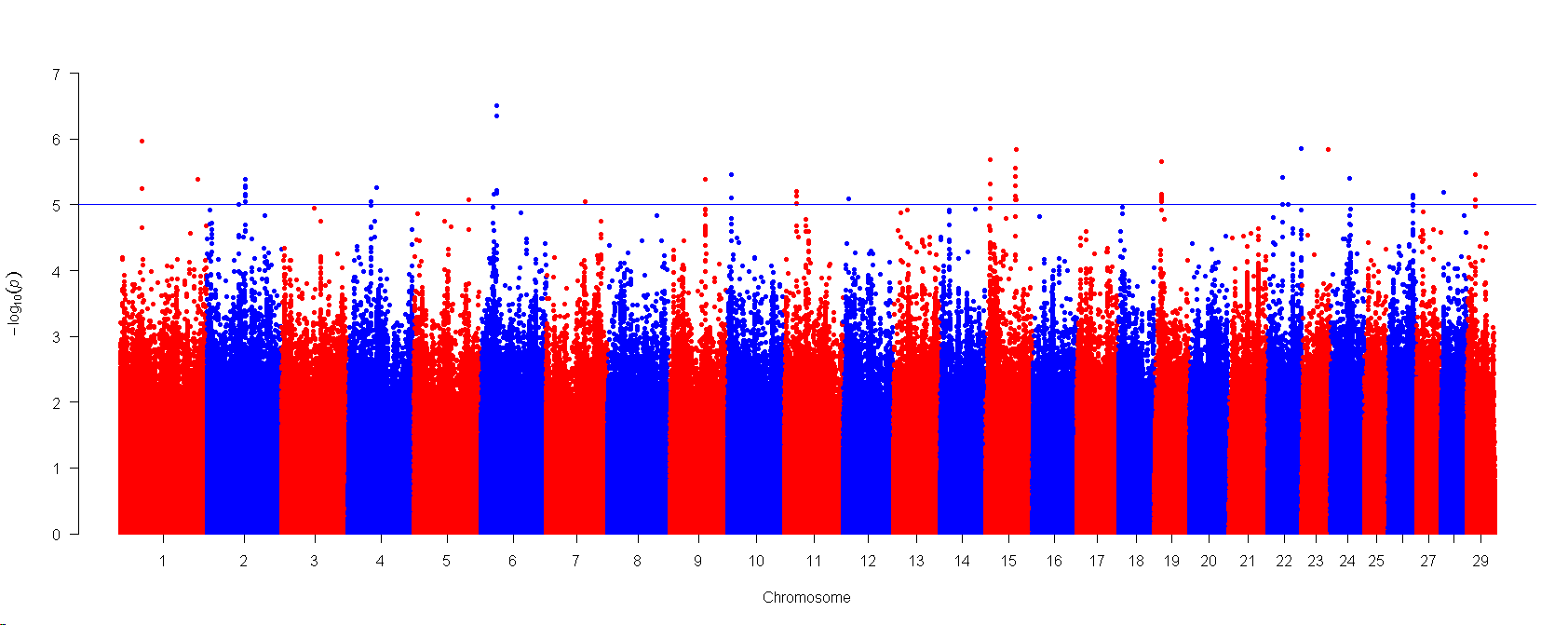 | 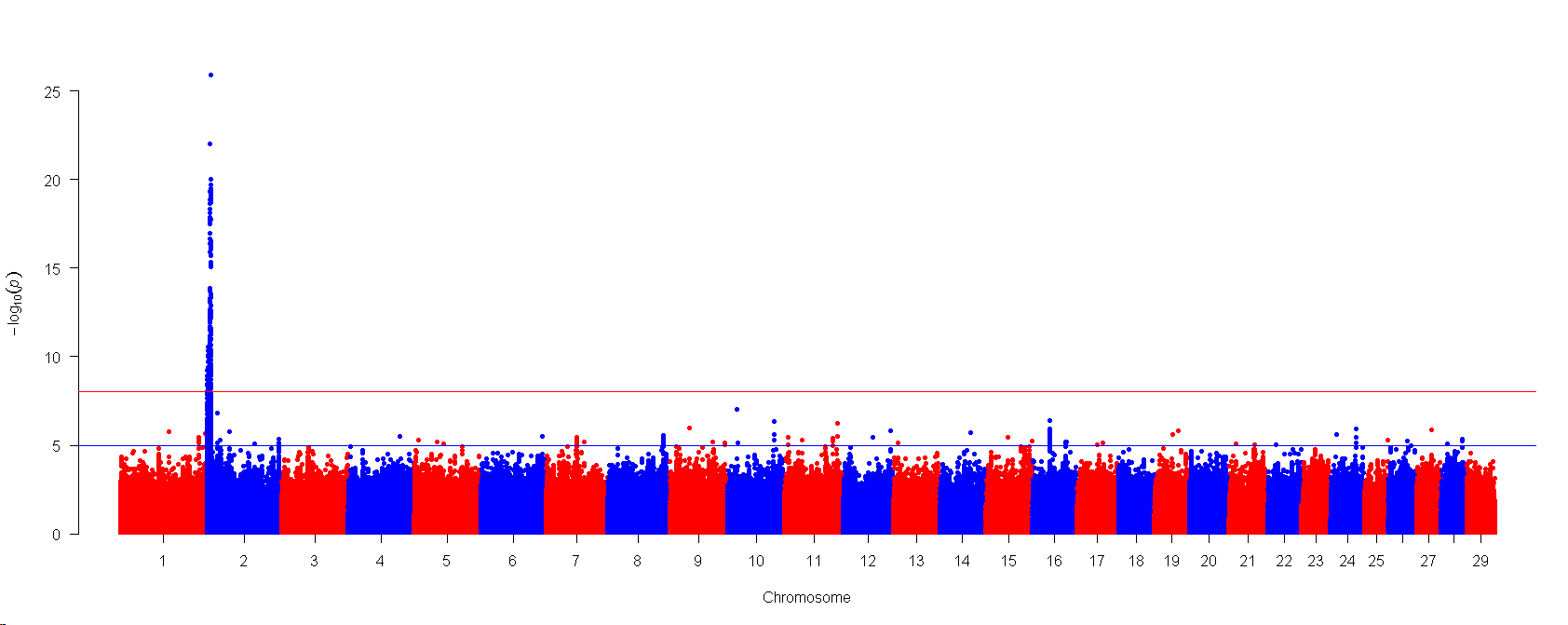 |  |
| **c)** | **d)** |  |
| 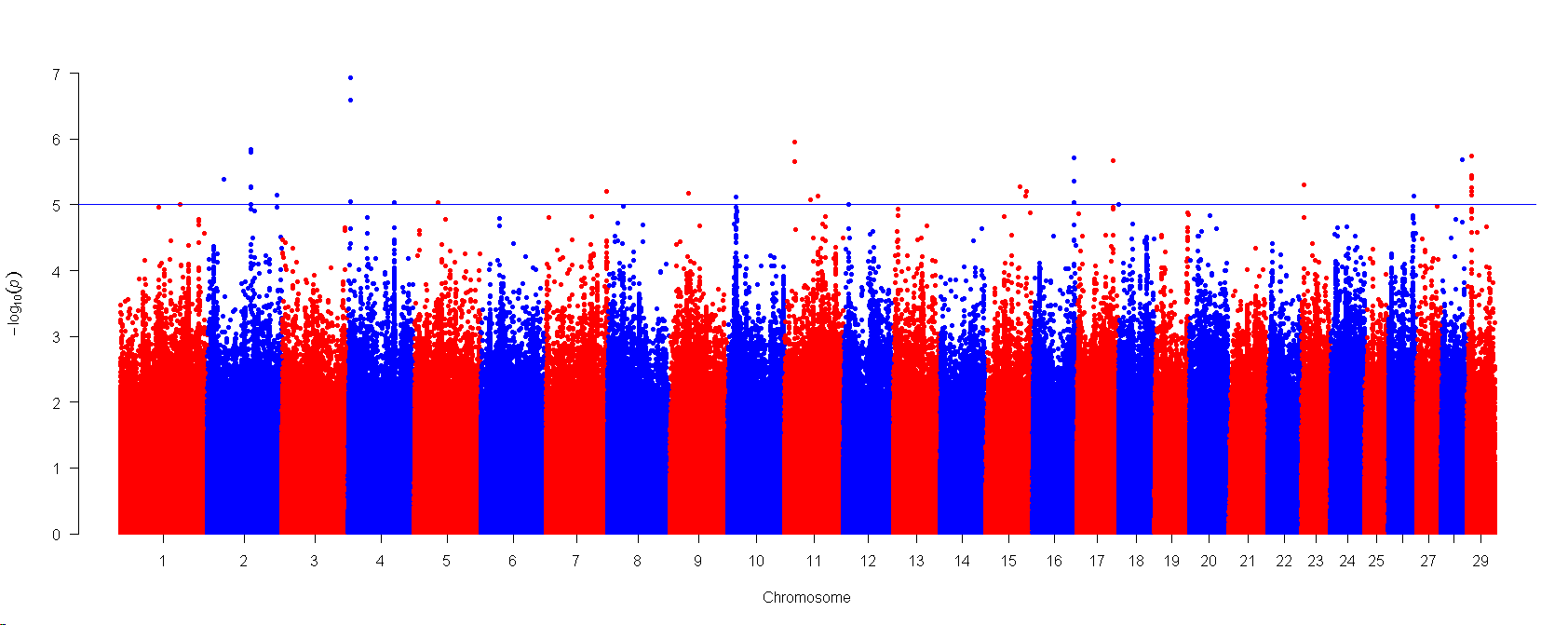 | 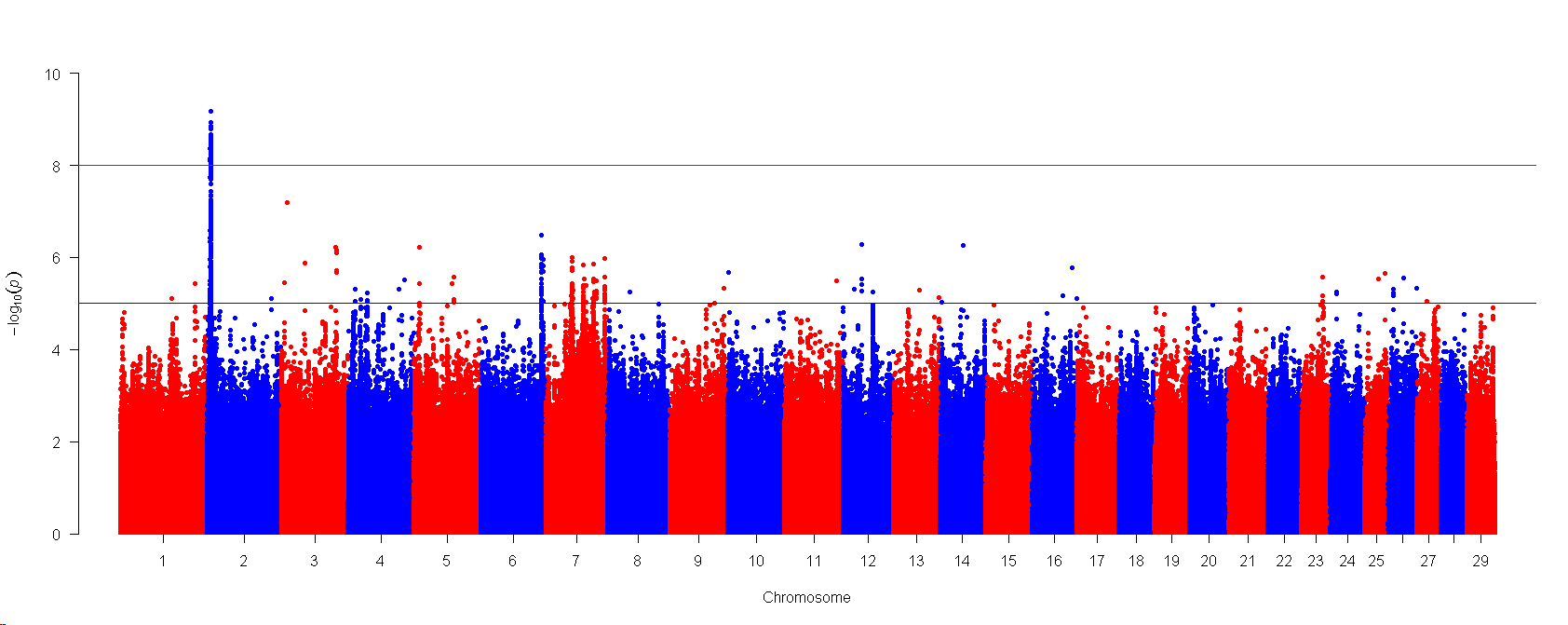 |  |
| **e)** | **f)** |  |
| 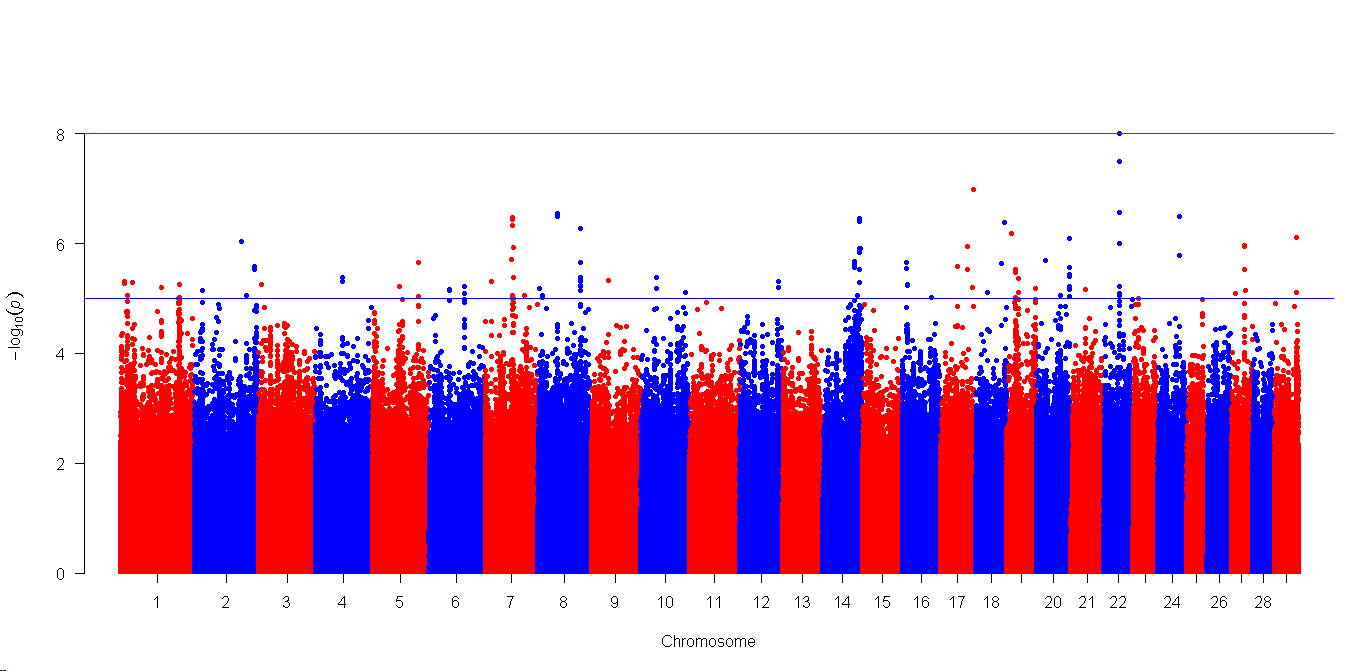 | 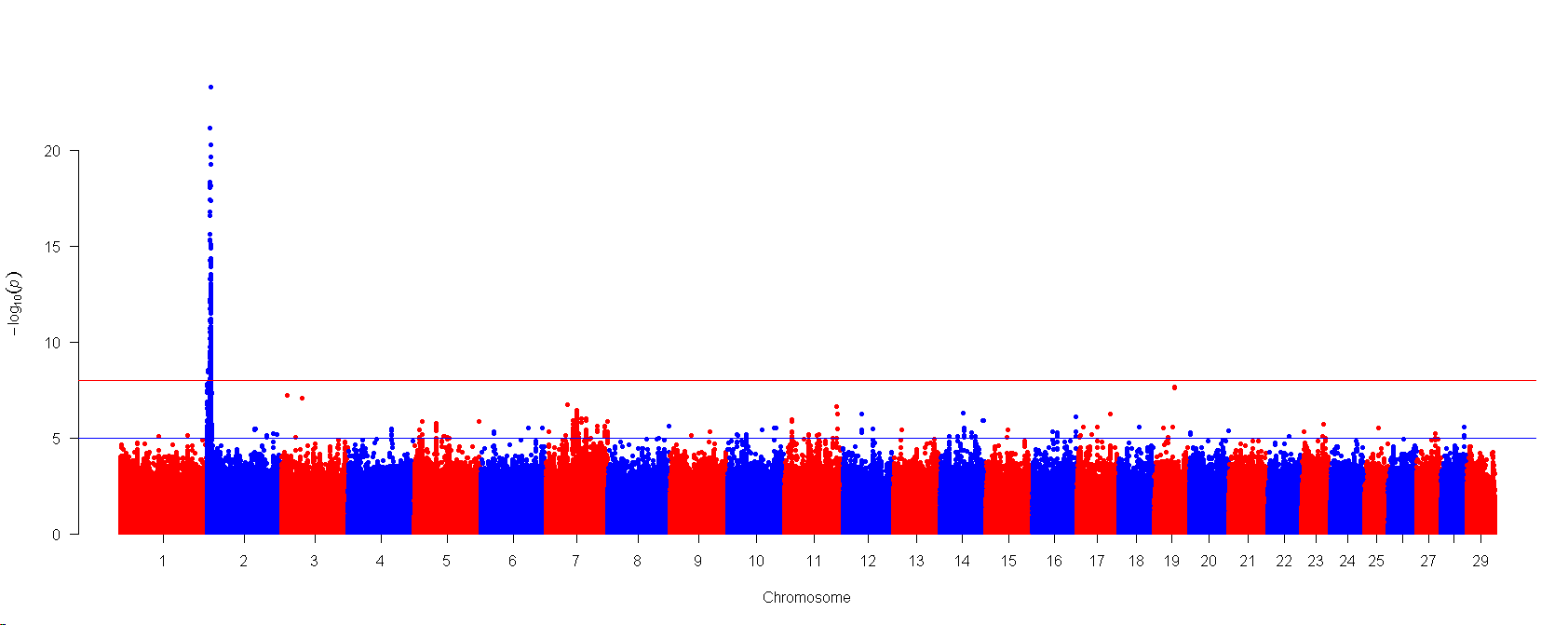 |  |

Figure S3: Manhattan plots for development of loin in a) Angus, b) Charolais, c) Hereford, d) Limousin, e) Simmental, and f) Meta-Analysis.

|  | **a)** | **b)** |
| --- | --- | --- |
| 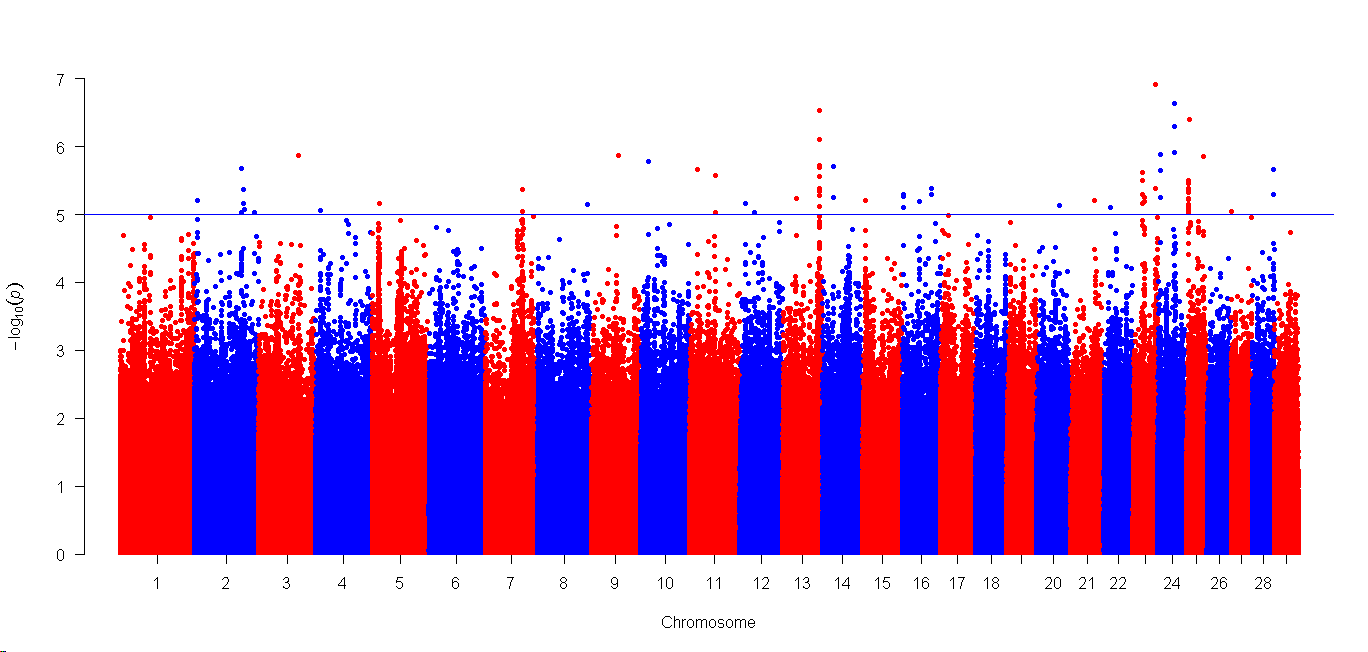 | 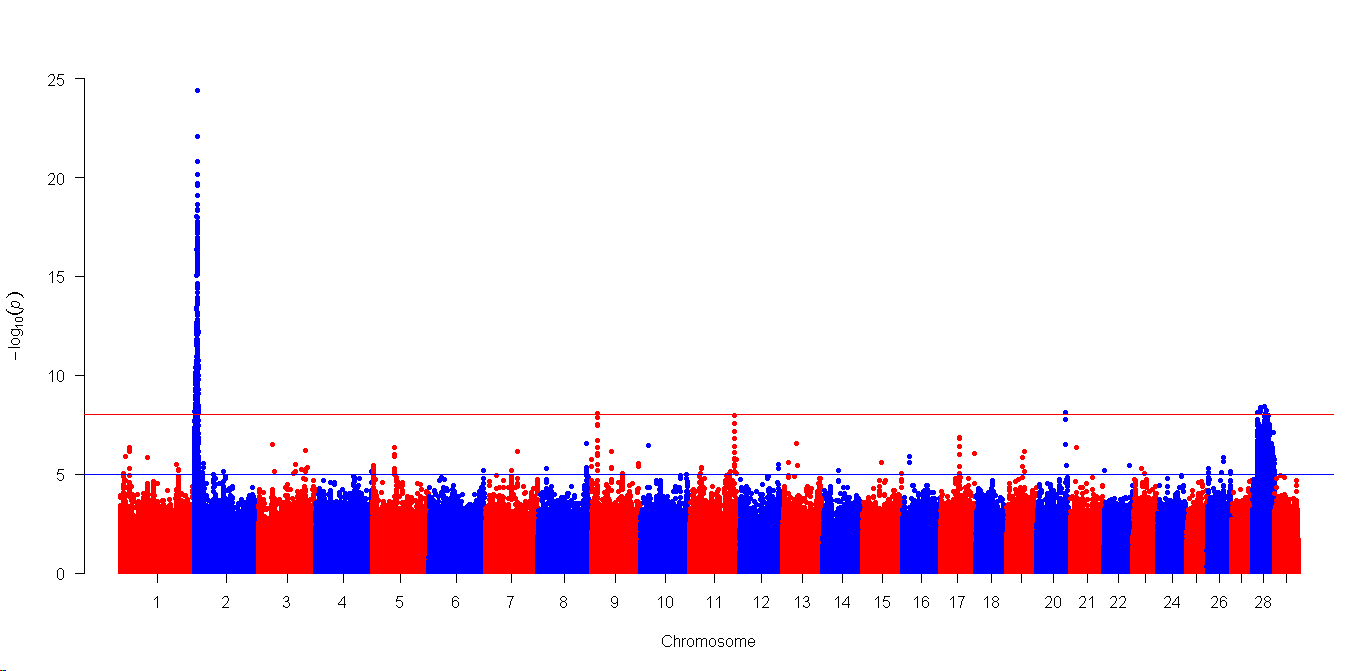 |  |
| **c)** | **d)** |  |
| 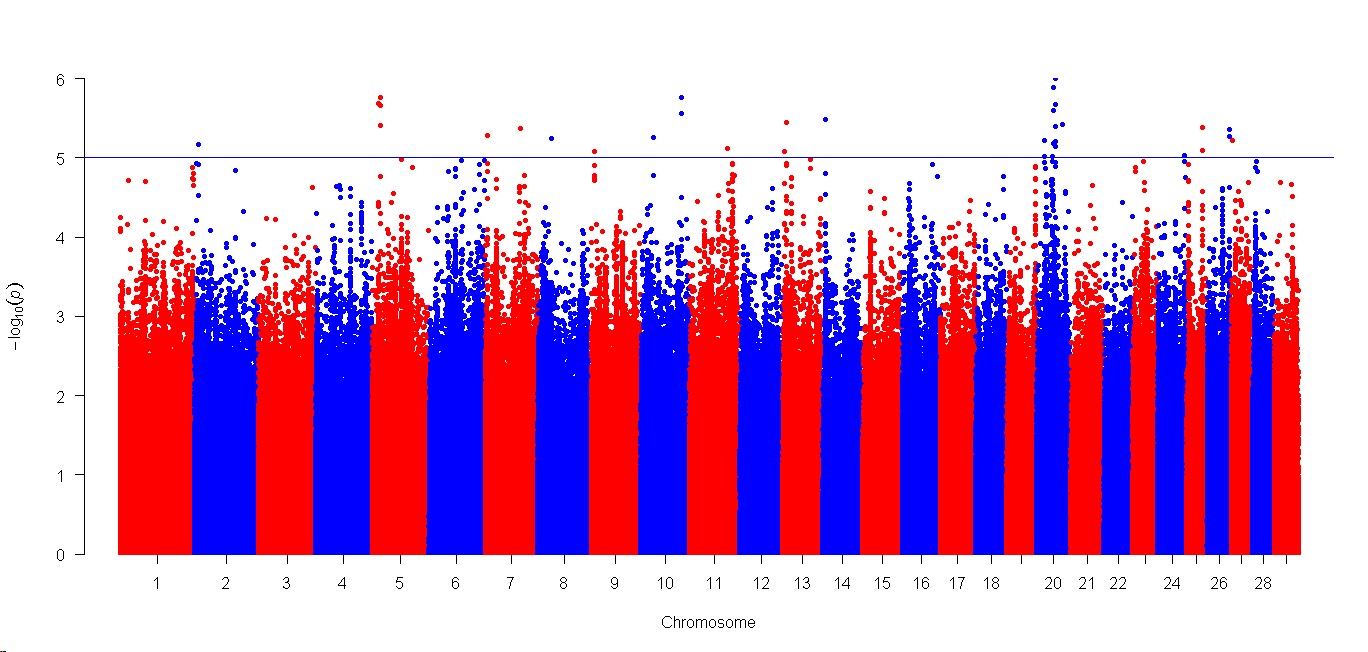 | 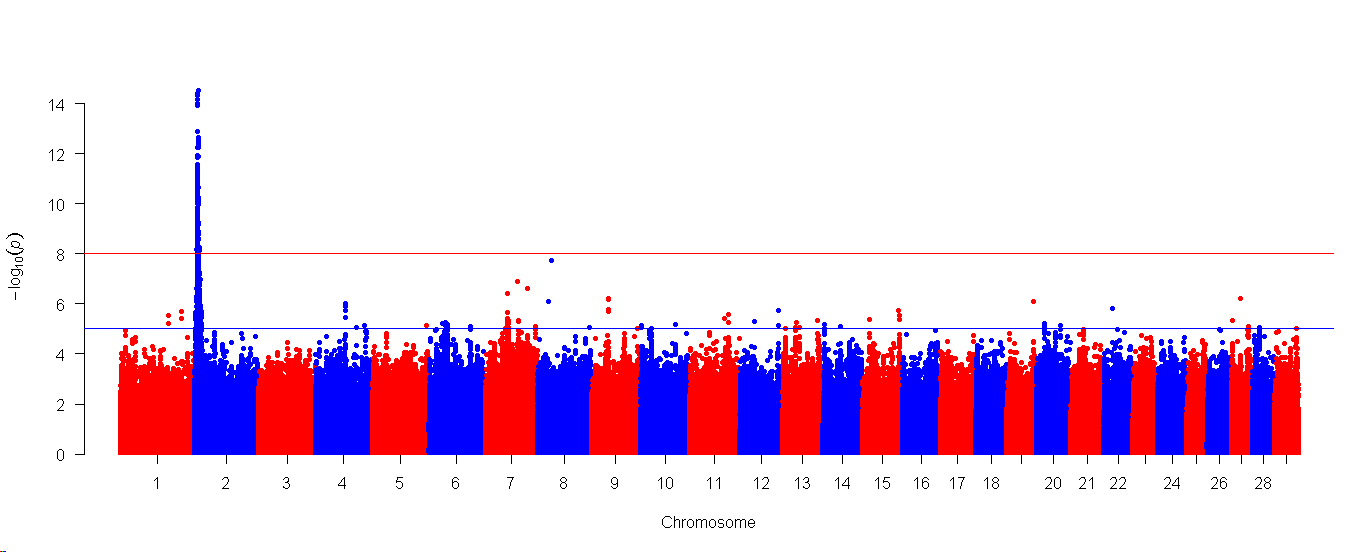 |  |
| **e)** | **f)** |  |
| 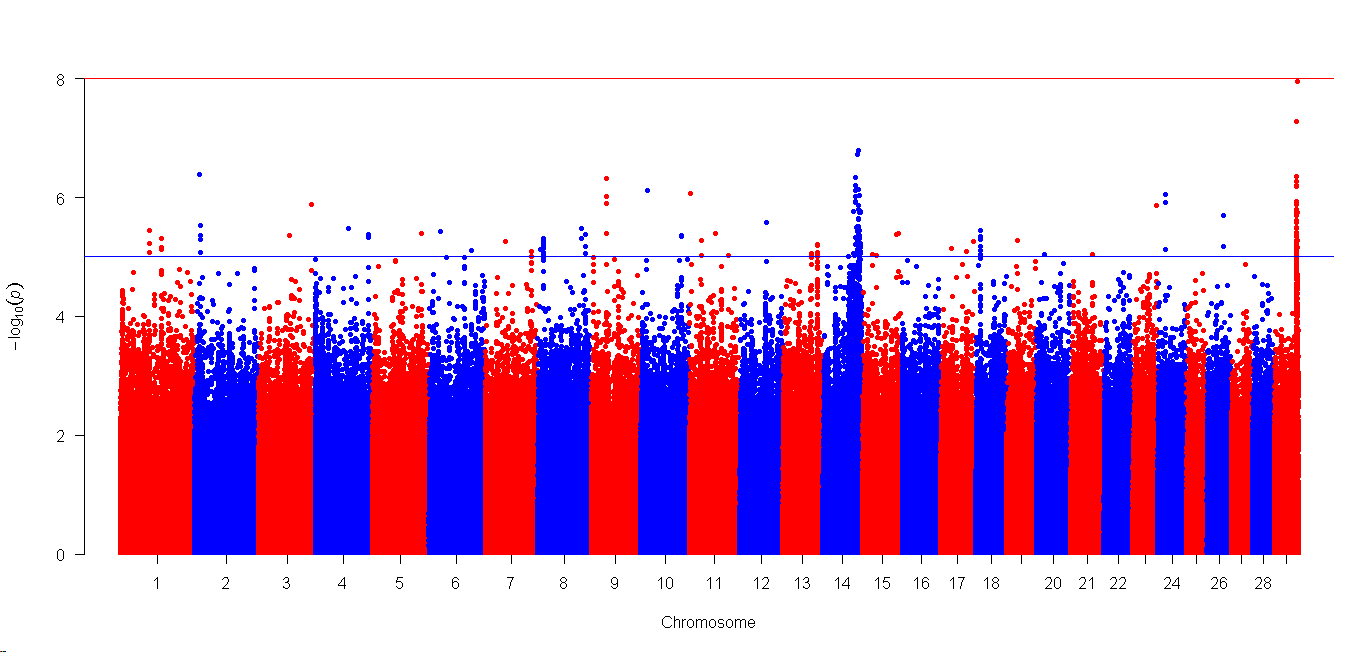 | 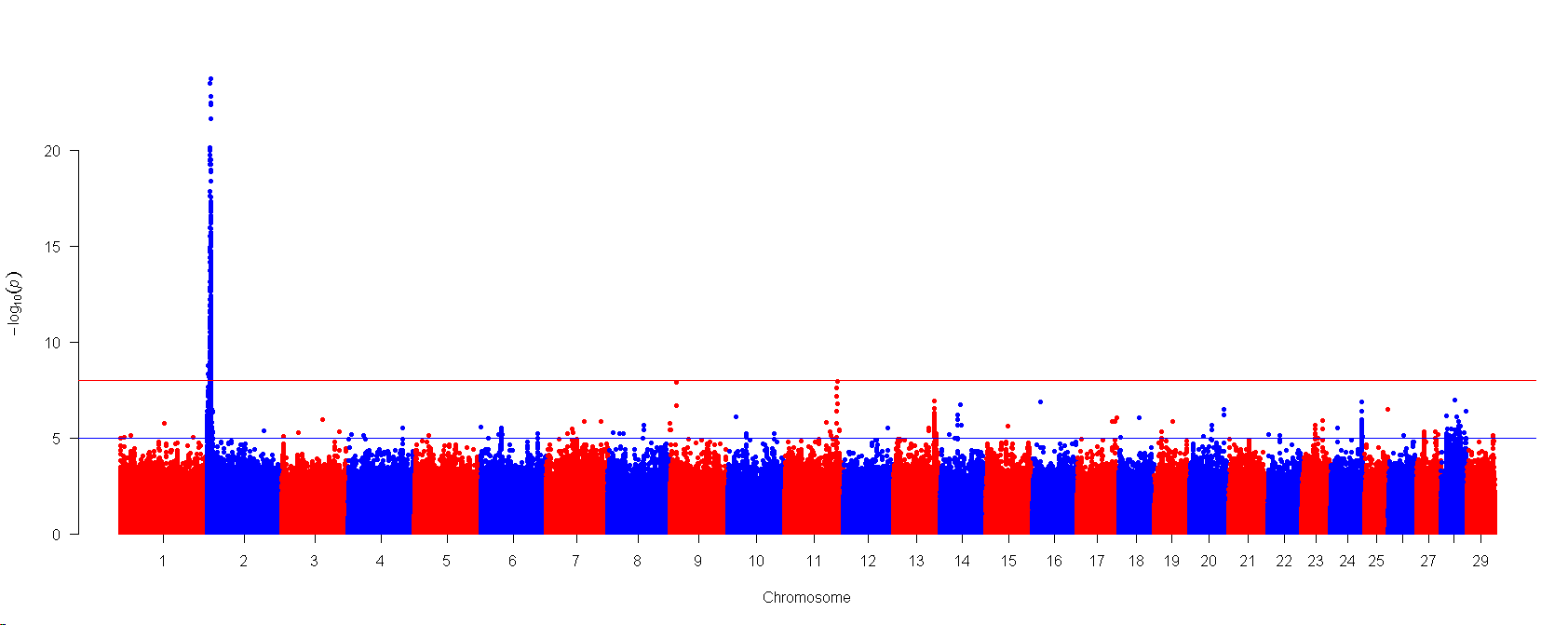 |  |

Figure S4: Manhattan plots for thigh width in a) Angus, b) Charolais, c) Hereford, d) Limousin, e) Simmental, and f) Meta-Analysis.

|  | **a)** | **b)** |
| --- | --- | --- |
| 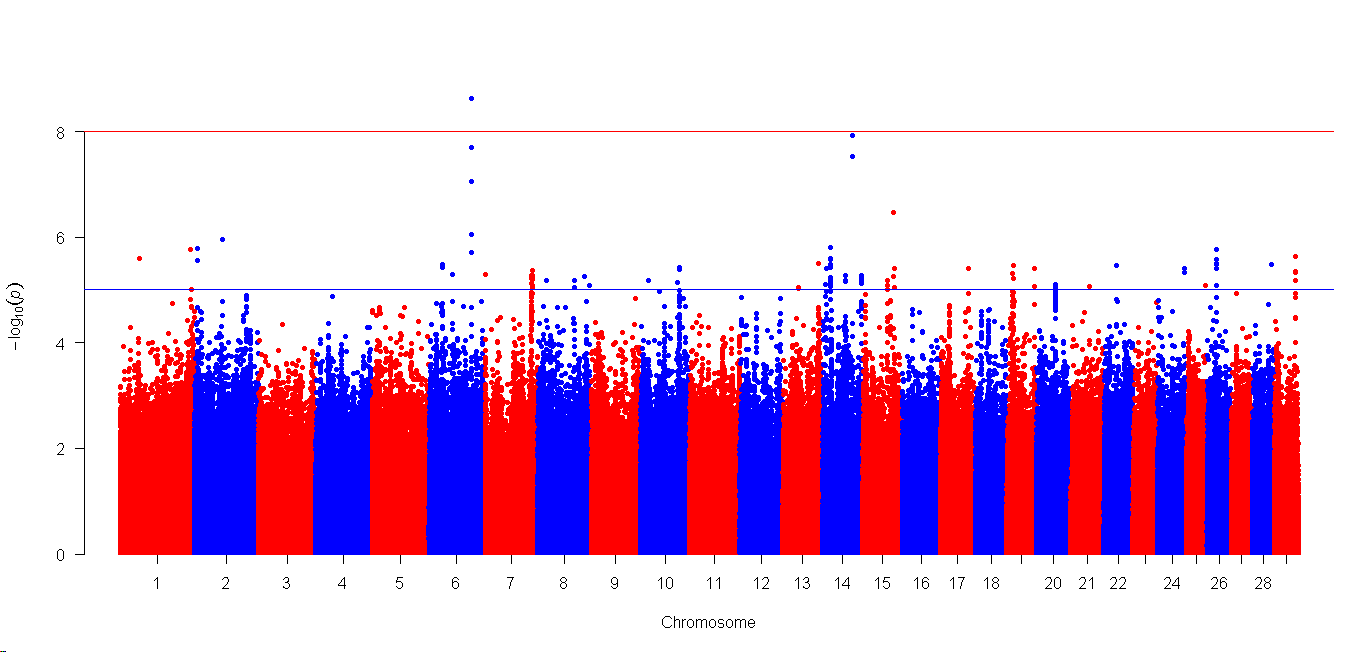 | 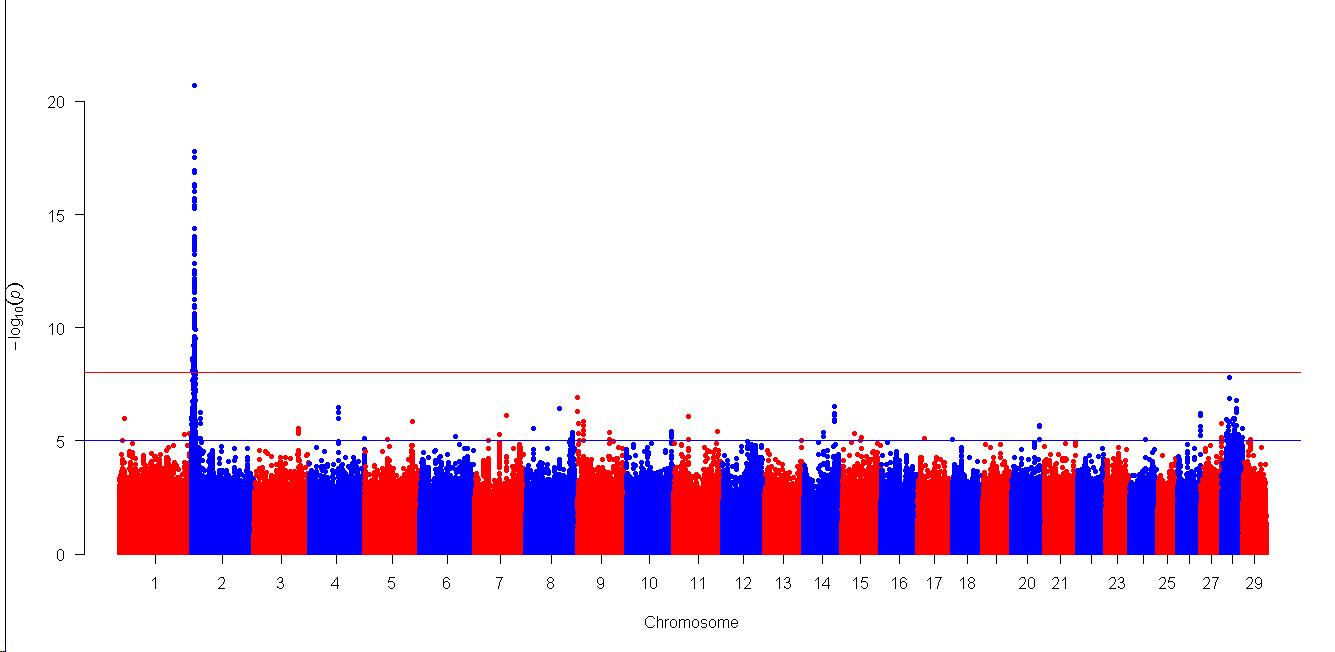 |  |
| **c)** | **d)** |  |
| 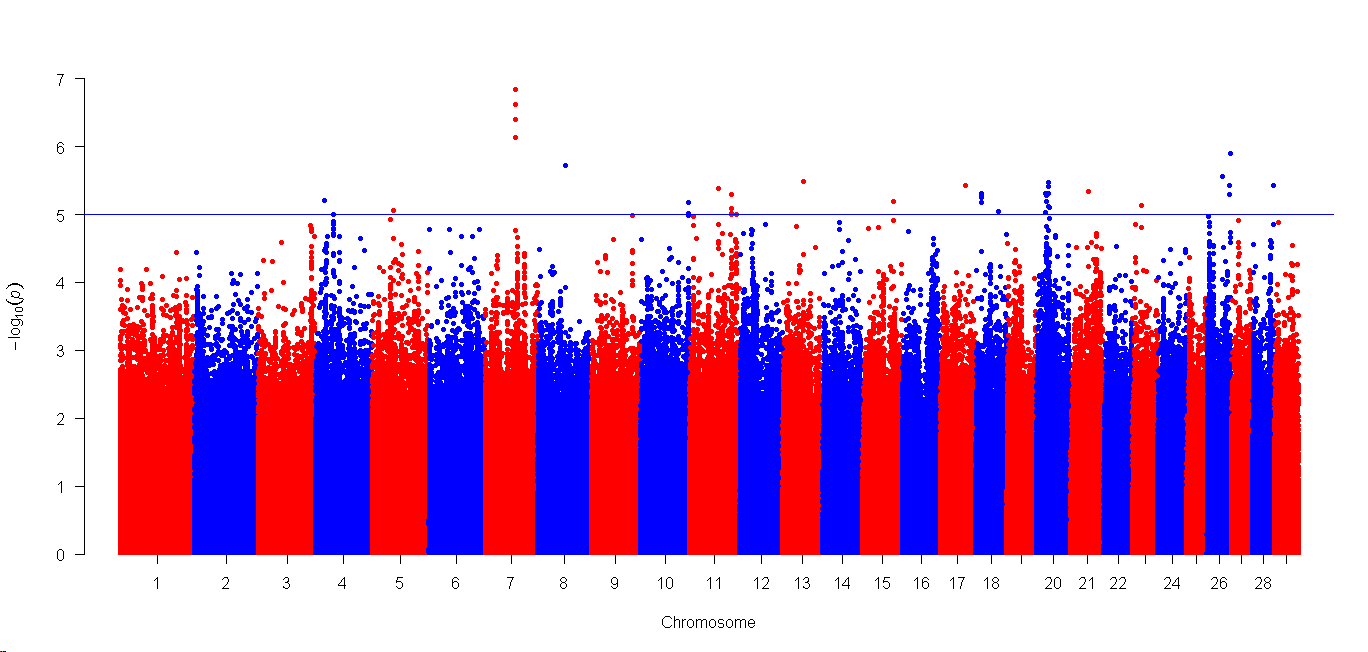 | 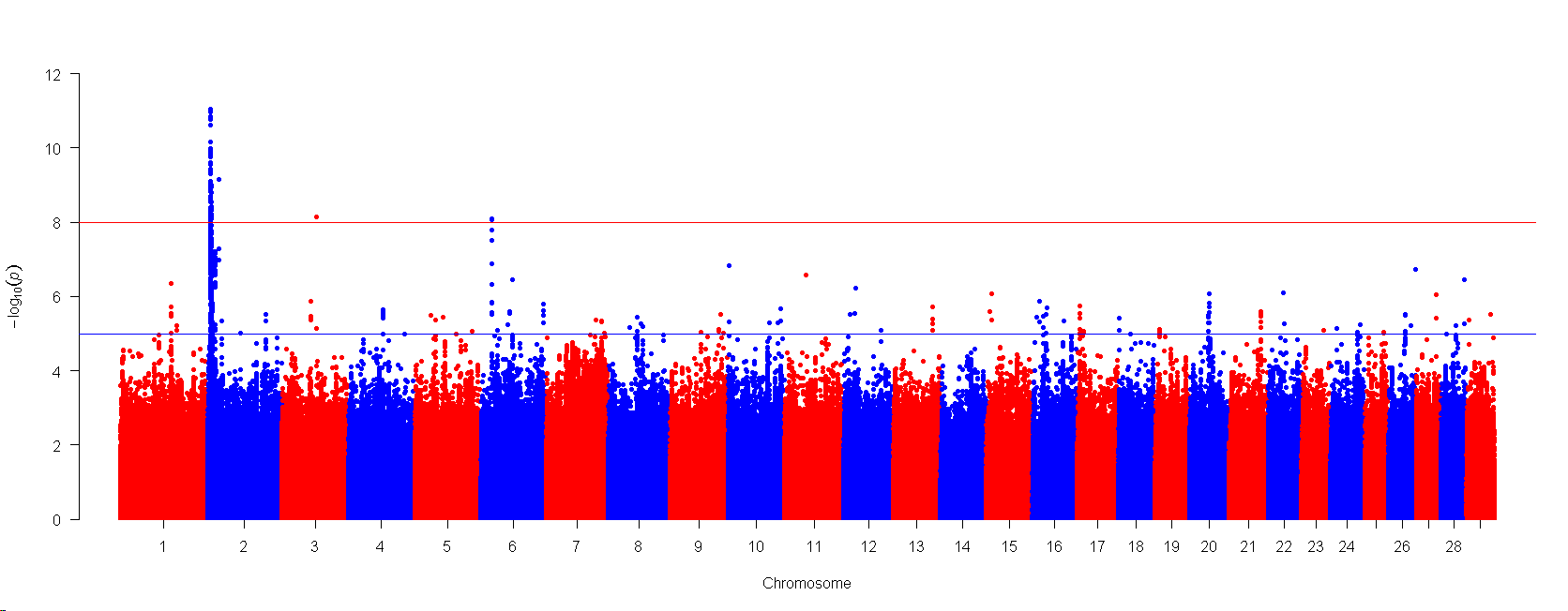 |  |
| **e)** | **f)** |  |
| 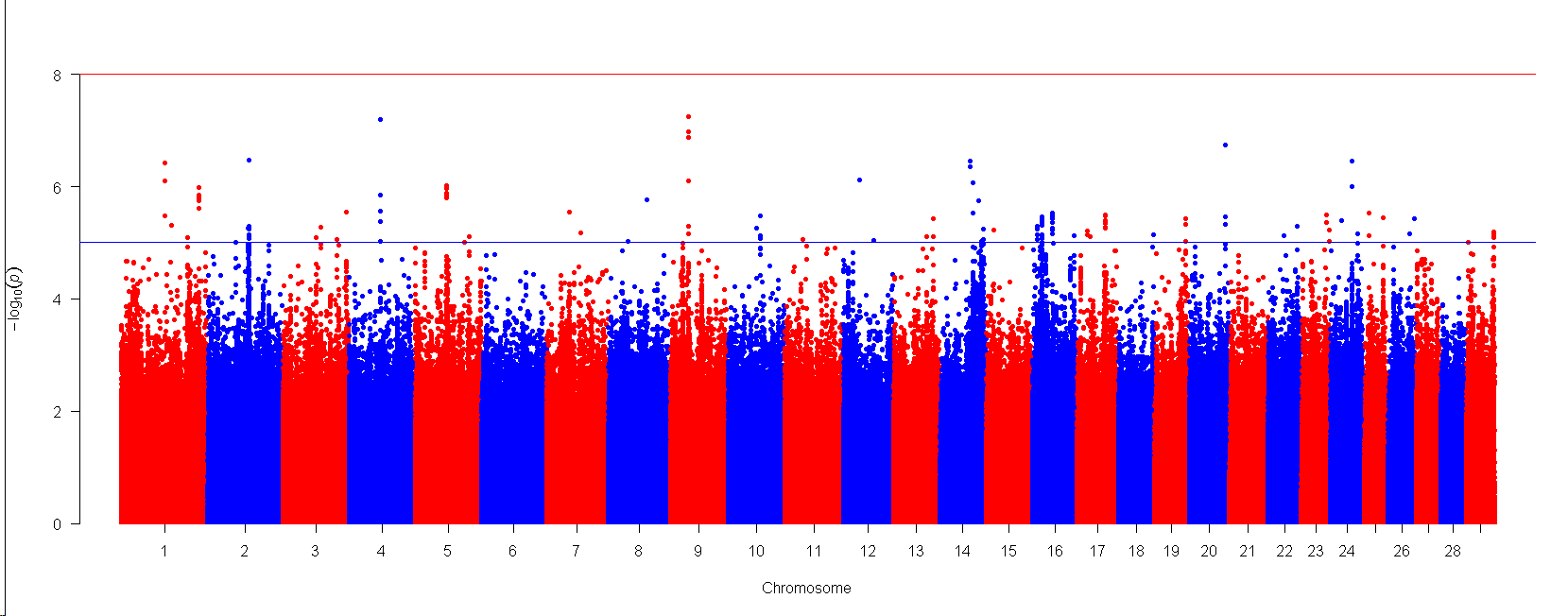 | 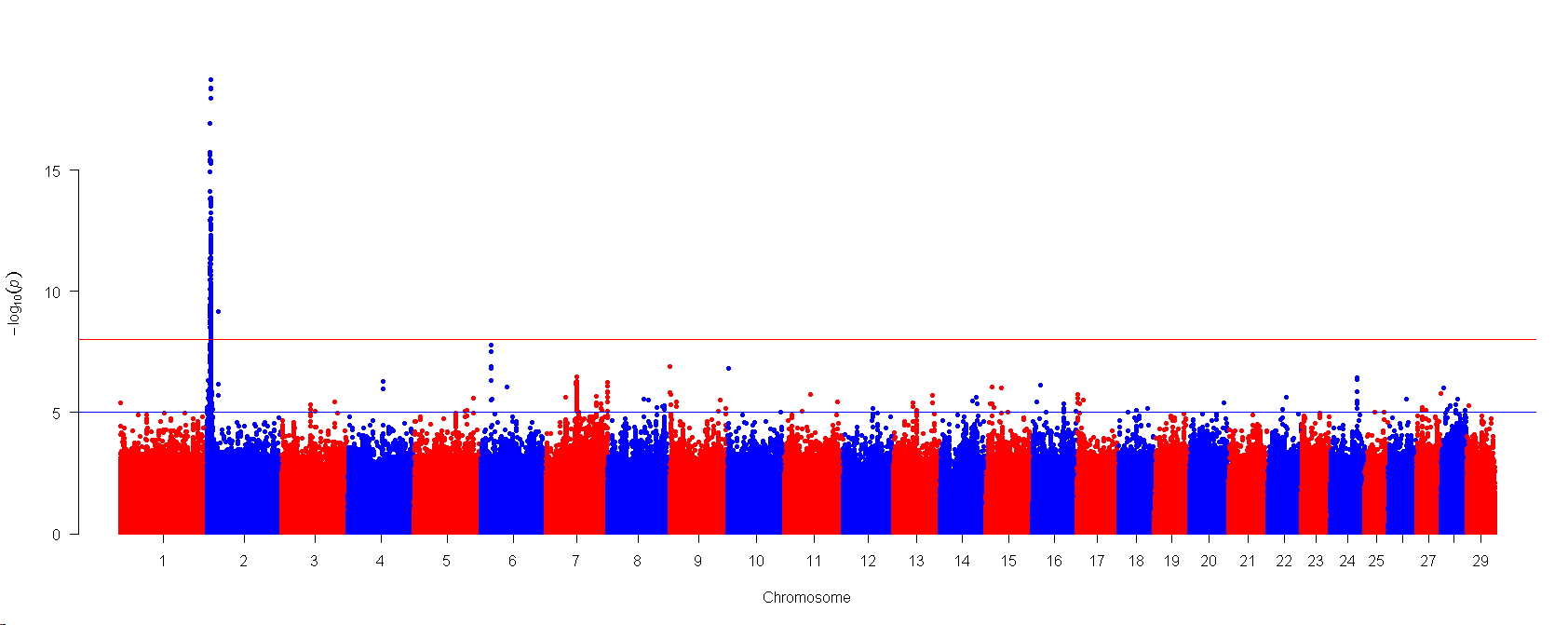 |  |

Figure S5: Manhattan plots for width of withers in a) Angus, b) Charolais, c) Hereford, d) Limousin, e) Simmental, and f) Meta-Analysis.
