# Supplementary material for: Genomic regions associated with muscularity in beef cattle differ in five contrasting cattle breeds
Source: Genet Sel Evol. 2020 Jan 30;52:2. doi: 10.1186/s12711-020-0523-1 (PMC6993462; doi:10.1186/s12711-020-0523-1)
Supplement: Supplementary file 3 — Additional file 3: Figure S6. Overlapping 1-kb regions that contain at least one suggestive or significant SNP for the five muscular traits in (a) Angus, (b) Charolais, (c) Hereford, (d) Limousin and (e) Simmental. Venn Diagram of overlapping 1-kb regions containing at least one suggestive or significant SNP in each of the five breeds. [file 12711_2020_523_MOESM3_ESM.docx]

| **a)** | | **b)** | |
| --- | --- | --- | --- |
| **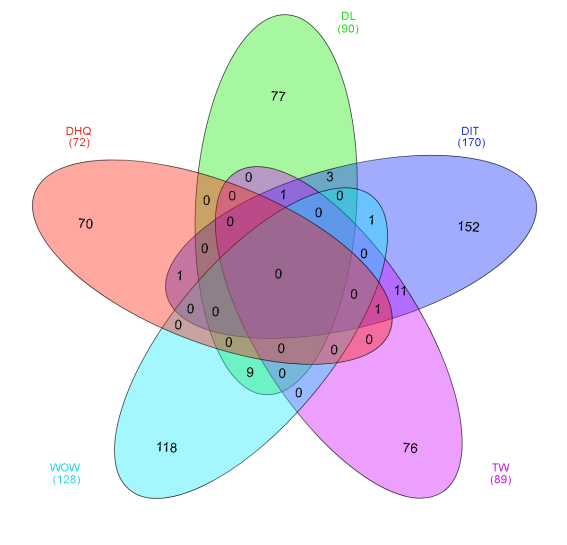** | | **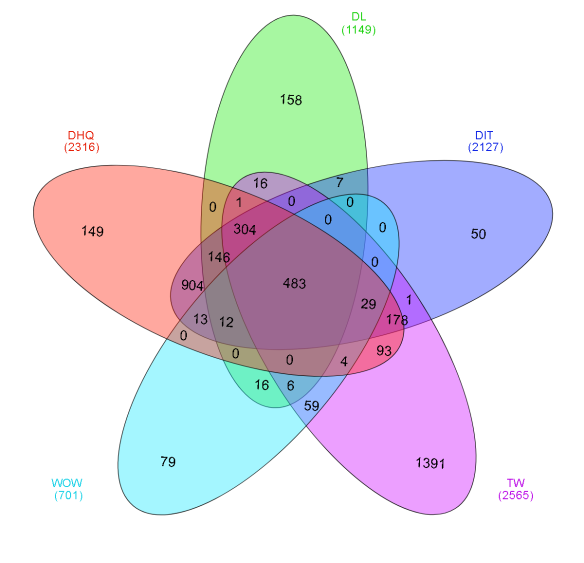** |  |
| **c)** | | **d)** |  |
| **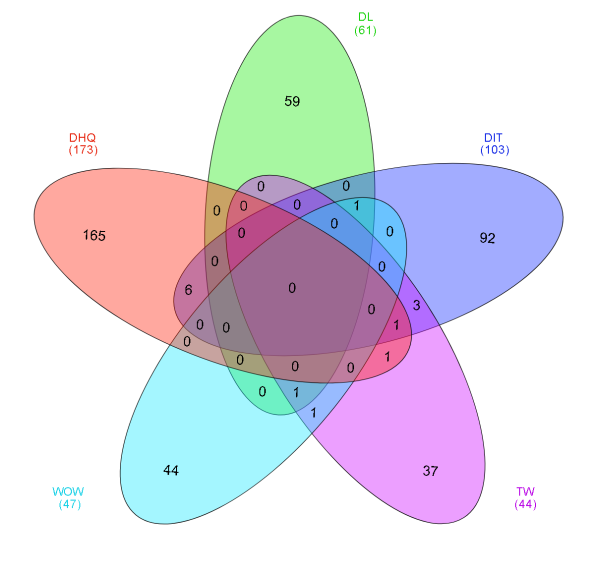** | | **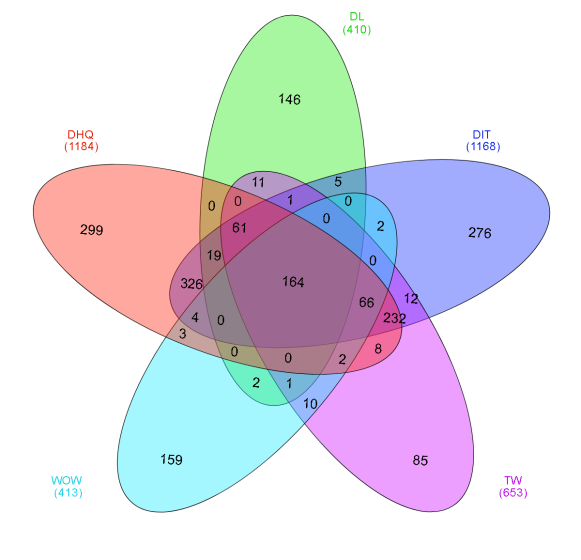** |  |
| **e)** | |  |  |
| 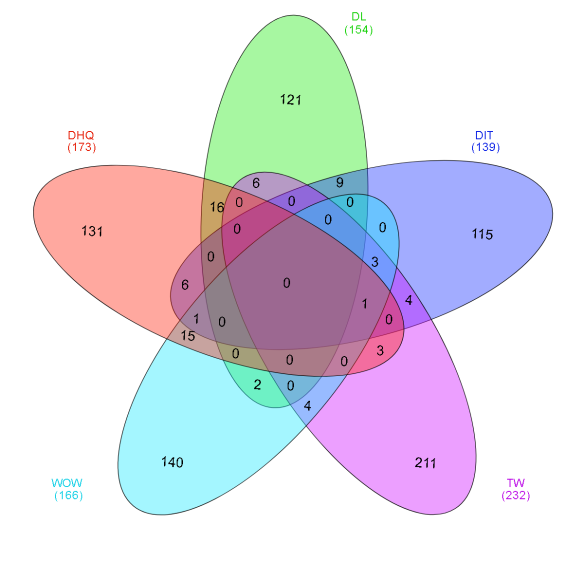 | |  |  |

Figure S6: Overlapping 1kb regions that contain at least one suggestive or significant SNP for the 5 muscular traits in a) Angus, b) Charolais, c) Hereford, d) Limousin and e) Simmental
