# Supplementary material for: Genomic regions associated with muscularity in beef cattle differ in five contrasting cattle breeds
Source: Genet Sel Evol. 2020 Jan 30;52:2. doi: 10.1186/s12711-020-0523-1 (PMC6993462; doi:10.1186/s12711-020-0523-1)
Supplement: Supplementary file 4 — Additional file 4. Location of the top five most significant QTL associated with each of the traits in the meta-analysis containing all five breeds. [file 12711_2020_523_MOESM4_ESM.docx]

Table S2: The location of the top 5 most significant QTLs associated with each of the traits in the meta-analysis containing all 5 breeds

|  |  |  |  | No of suggestive and significant SNPs | Most significant SNP |  | Candidate genes within this QTL |
| --- | --- | --- | --- | --- | --- | --- | --- |
| Trait | Chr | Start | End |  |  | P-Value |  |
| DHQ | 2 | 35194 | 12618550 | 3545 | 6630781^a^ | 2.06x10^-47^ | WDR75^, ASNSD1^, ARHGEF4*, MYO7B^, NAB1^, MFSD6^, MSTN^, PMS1^, ORMDL1*, COL3A1^, COL5A2^, ANKAR^, SLC40A1 |
|  | 4 | 73026098 | 74288232 | 3 | 73526098^b^ | 8.36x10^-7^ | ZNF804B*, TEX47 |
|  | 5 | 59612855 | 60677712 | 6 | 60112855^a^ | 1.46E-07 | AMDHD1, TESPA1, NTN4, SNRPF |
|  | 8 | 65991701 | 67324054 | 5 | 66811769^a^ | 1.45x10^-6^ |  |
|  | 14 | 33353270 | 34360874 | 6 | 33853270^a^ | 5.37x10^-7^ | ARFGEF1, PREX2 |
| DIT | 2 | 156108 | 11096370 | 3358 | 6808074^a^ | 2.22x10^-44^ | WDR75^, ASNSD1^, ARHGEF4*, MYO7B^, NAB1^, MFSD6^, MSTN^, PMS1^, ORMDL1*, COL3A1^, COL5A2^, ANKAR^, SLC40A1^ |
|  | 9 | 7672322 | 8675817 | 6 | 8173505^b^ | 3.13x10^-7^ | ADGRB3* |
|  | 13 | 7208015 | 7341939 | 5 | 7708015^a^ | 1.85x10^-7^ | TASP1 |
|  | 13 | 46254146 | 47297509 | 37 | 46754146^a^ | 4.36x10^-7^ | IDI1, GTPBP4 |
|  | 14 | 33063362 | 34360874 | 3 | 33853270^a^ | 5.61x10^-8^ | ARFGEF1, PREX2 |
| TW | 2 | 35194 | 10589284 | 1806 | 6808074^a^ | 1.85x10^-24^ | WDR75^, ASNSD1*, ARHGEF4, MYO7B*, NAB1*, MFSD6*, MSTN^, PMS1^, ORMDL1, COL3A1*, COL5A2*, ANKAR*, SLC40A1^ |
|  | 11 | 94484006 | 96696774 | 8 | 96162620^b^ | 1.09x10^-8^ | LHX2, PSMB7, WDR38, GOLGA1 |
|  | 13 | 74868306 | 75902585 | 94 | 75384004^b^ | 1.20x10^-7^ | DNTTIP1, TNNC2, ACOT8 |
|  | 24 | 56615639 | 57674865 | 21 | 57144827^a^ | 1.37x10^-7^ | WDR7, FECH |
|  | 28 | 24372370 | 25568836 | 5 | 25068836^c^ | 1.21x10^-7^ | SIRT1, MYPN, DNA2, SLC25A16 |
| DL | 2 | 167014 | 9934770 | 845 | 6808074^a^ | 5.51x10^-24^ | WDR75^, ASNSD1*, ARHGEF4, MYO7B*, NAB1*, MFSD6*, MSTN^, PMS1^, ORMDL1, COL3A1^, COL5A2^, ANKAR^, SLC40A1^ |
|  | 7 | 55126795 | 56162374 | 82 | 55642210^a^ | 3.49x10^-7^ | SPRY4, FGF1 |
|  | 12 | 32803688 | 33961156 | 5 | 33461156^b^ | 6.02x10^-7^ | USP12, SHISA2 |
|  | 16 | 79153910 | 80205278 | 3 | 79653910^a^ | 7.65x10^-7^ | ATP6V1G3, PTPRC |
|  | 19 | 36066854 | 37067996 | 6 | 36567996^a^ | 2.22x10^-8^ | TOB1, WFIKKN2, MYCBPAP |
| WOW | 2 | 1889616 | 8714844 | 735 | 6727404^b^ | 1.86x10^-19^ | WDR75^, ASNSD1*, ARHGEF4, MYO7B*, NAB1, MFSD6, MSTN^, PMS1^, ORMDL1, COL3A1^, COL5A2^, ANKAR^, SLC40A1^ |
|  | 6 | 19014612 | 20892119 | 10 | 19708641^a^ | 1.63x10^-8^ | NPNT, GSTCD |
|  | 7 | 55104801 | 56162374 | 90 | 55628366^a^ | 3.40x10^-7^ | SPRY4, FGF1* |
|  | 9 | 69711 | 2115412 | 3 | 618653^a^ | 1.25x10^-7^ | PTP4A1, PHF3 |
|  | 24 | 49067824 | 50114190 | 28 | 49567824^b^ | 3.78E-07 | SMAD7, ACAA2, MYO5B |

Superscript denotes SNP classification: ^a^intergenic, ^b^intron, ^c^upstream gene variant. Symbols denote the significance of SNPs within genes: *gene contained at least one suggestive SNP, ^ gene contained at least one significant SNP.
